# Exploring happiness factors with explainable ensemble learning in a global pandemic

**DOI:** 10.1371/journal.pone.0313276

**Published:** 2025-01-02

**Authors:** Md Amir Hamja, Mahmudul Hasan, Md Abdur Rashid, Md Tanvir Hasan Shourov

**Affiliations:** 1 Department of Statistics, Hajee Mohammad Danesh Science and Technology University, Dinajpur, Bangladesh; 2 Department of Computer Science and Engineering, Hajee Mohammad Danesh Science and Technology University, Dinajpur, Bangladesh; 3 Department of Sociology, Hajee Mohammad Danesh Science and Technology University, Dinajpur, Bangladesh; Atlantic Technological University, IRELAND

## Abstract

Happiness is a state of contentment, joy, and fulfillment, arising from relationships, accomplishments, and inner peace, leading to well-being and positivity. The greatest happiness principle posits that morality is determined by pleasure, aiming for a society where individuals are content and free from suffering. While happiness factors vary, some are universally recognized. The World Happiness Report (WHR), published annually, includes data on ‘GDP per capita’, ‘social support’, ‘life expectancy’, ‘freedom to make life choices’, ‘generosity’, and ‘perceptions of corruption’. This paper predicts happiness scores using Machine Learning (ML), Deep Learning (DL), and ensemble ML and DL algorithms and examines the impact of individual variables on the happiness index. We also show the impact of COVID-19 pandemic on the happiness features. We design two ensemble ML and DL models using blending and stacking ensemble techniques, namely, Blending RGMLL, which combines Ridge Regression (RR), Gradient Boosting (GB), Multilayer Perceptron (MLP), Long Short-Term Memory (LSTM), and Linear Regression (LR), and Stacking LRGR, which combines LR, Random Forest (RF), GB, and RR. Among the trained models, Blending RGMLL demonstrates the highest predictive accuracy with *R*^2^ of 85%, MSE of 0.15, and RMSE of 0.38. We employ Explainable Artificial Intelligence (XAI) techniques to uncover changes in happiness indices, variable importance, and the impact of the COVID-19 pandemic on happiness. The study utilizes an open dataset from the WHR, covering 156 countries from 2018 to 2023. Our findings indicate that ‘GDP per capita’ is the most critical indicator of happiness score (HS), while ‘social support’ and ‘healthy life expectancy’ are also important features before and after the pandemic. However, during the pandemic, ‘social support’ emerged as the most important indicator, followed by ‘healthy life expectancy’ and ‘GDP per capita’, because social support is the prime necessity in the pandemic situation. The outcome of this research helps people understand the impact of these features on increasing the HS and provides guidelines on how happiness can be maintain during unwanted situations. Future research will explore advanced methods and include other related features with real-time monitoring for more comprehensive insights.

## Introduction

The meaning of happiness can vary from person to person. But it has a significant role in our lives and can have a great impact on the way we live. Oxford English dictionary defines happiness as a state of feeling or showing pleasure. Happiness can be characterized as a persistent mental state that encompasses not just emotions such as joy, contentment, and other positive feelings but also a perception that one’s life possesses significance and worth [1]. There are four dimensions of happiness that’s are pleasure, engagement, meaning, and balanced life [2, 3]. Various research has indicated that happiness has benefits of a wider range [4]. Physicians who experience happiness are inclined to reach quicker and more precise diagnoses, as suggested by multiple studies [5]. Not only this, the learning ability of students also increases due to happiness during the learning period [6]. In addition to the mentioned individual advantages, individuals who experience greater happiness also enjoy improved well-being and extended lifespans [7], exhibit safer behavior while driving, reduce the likelihood of accidents [8], and contribute positively to society as a whole [9]. Recognizing the significance of happiness, the United Nations Sustainable Development Solutions Network has released World Happiness Reports (WHRs), ranking countries based on their happiness levels since 2012 [10]. The happiness index, also known as the Happiness Score (HS) or ladder score or well-being index, is a measurement used to assess and quantify the level of happiness or well-being within a population or country. These reports aim to explore happiness by considering factors like Gross Domestic Product (GDP) per capita, social support, life expectancy, perception of corruption, freedom to make life choices, and generosity. The index provides a comparative measure of happiness across different regions or nations and is often used to guide policy decisions and prioritize well-being initiatives [10].

Comparing the WHR rankings of pre-pandemic (2018-2019) and post-pandemic (2022-2023), there is a combination of consistency and change. While eight out of the top 10 countries in 2023 were also in the top 10 in 2018, the specific rankings shifted. Finland emerged as the leader, closely followed by Denmark and Iceland, with average scores between 7.78 and 7.52. Norway made a significant downfall from third place in 2018 to claim the eighth spot in 2023. Luxembourg and Israel make a significant improvement in the Happiness Index to get involved in the top ten countries. However, Canada and Austria failed to maintain their position in the top ten countries. Since predicting HS of countries is an important task for researchers as well as countries various techniques have been used to predict world HS over the past decades. Statistical approaches, econometric approaches, Machine Learning (ML), and Deep Learning (DL) approaches have been utilized to model the intrinsic complexity of the prediction. Most of the studies used Deep Neural Networks (DNN) [11–13], Decision Tree (DT) [13–15]and Support Vector Machines (SVM) [11, 16] to predict the HS for different countries. The unsupervised learning such as clustering [17] have also been used to predict this score. Particular attention is increasingly being paid to DL, such as Multilayer Perceptron (MLP) and DNN are also used for predicting happiness [13, 14]. Those techniques have a great impact in happiness score prediction but due to COVID-19 pandemic, the individual features value changes in an unorganized patterns that create the problems to analyze the HS based on the current models. The coronavirus, having infected numerous individuals, has escalated into a pandemic. Reports suggest that the total number of deaths during the COVID-19 pandemic in 2020 could exceed 3 million, surpassing the official count by 1.2 million [18]. Amidst the COVID era, many have endured job losses, which indirectly contribute to feelings of sadness. Presently, people prioritize more than just financial stability; they also value aspects such as quality of life, mental well-being, and other related concerns. Programs like “Art of Living” have gained traction as happiness becomes a prominent topic [19]. The COVID-19 period proved to be mentally exhausting for many individuals. There is a strong inclination to understand the factors contributing to the significant decline in mental health during this time. As mentioned earlier, the values of the features changes dramatically in the COVID-19 that create an impact on the models performances thus the story behind this change need to be analyze. Its motivate us to employ advance techniques like Explainable Artificial Intelligence (XAI) models to show the story behind the black-box ML and DL models. By leveraging XAI models, we can gain insights into the factors that significantly impact mental well-being during a pandemic. Many previous studies focused solely on predicting national HS, our study takes a novel approach by implementing XAI techniques. By doing so, we aim to identify the factors that can affect national HS. This approach provides a deeper understanding of how these factors interact and work together to influence the HS of countries, thereby contributing to a more comprehensive analysis of happiness determinants. Additionally, to get the better prediction, our study also propose two ensemble model that have the capability to predict the score more accurately than existing models. The core contributions of this study are below:

- To predict Happiness Scores (HS) using World Happiness Report (WHR) data from 2018-2023, we design a system driven by ML, DL, Ensemble Learning, and Explainable AI. This system not only predicts national HS but also provides model explainability to reveal the underlying factors behind the predictions.
- To get the better performance of the models in HS prediction, we develop two ensemble models using stacking and blending ensemble technique and compare their performance with existing ML and DL models to find the superiority of the proposed models.
- To find the behind story of national HS and the impact of the individual features on model performances, we use LIME, SHAP, ELI5 XAI techniques. The methods shows the global and local explainability together on the datasets, where we identify the most important factors, their behaviours, change in patters and future trend.
- To examine the impact of the COVID-19 pandemic in HS, we focus on the track change in the ranking of the features and analyze how each feature influences the HS along with the model’s performance.

The rest of the paper organized as in the Literature Review section the related literature’s, in the Materials and Methods section the details methods, the findings are in the Results and Discussion section and finally in Section Conclusion the conclusion and future research directions are described.

## Literature review

This section provides an overview of prior research concerning the prediction of HS. It outlines existing methodologies and recent advancements in predicting HS. Current researches suggest that happiness serves as a vital indicator of societal well-being [20], aligning with Betham’s assertion that the best society prioritizes citizen happiness [21]. Numerous studies have explored the positive aspects of happiness in policy making [22, 23]. However, manual analysis of the factors influencing happiness is both labor-intensive and costly. Hence, there is a growing need for automatic analysis to address the complexity and expense associated with this task. A decent paper in 2020 “Well-being is more than happiness and life satisfaction” analyzes the relationships between happiness and well-being across 21 countries [24]. It distinguishes between happiness and various components like competence, emotional stability, positive emotions, and engagement. Unlike previous studies, it identifies separate dimensions of positivity and also find that while GDP and similar factors are related to happiness, they are not entirely correlated. In 2011, Louise Millard employs various ML techniques to analyze global happiness, utilizing Principal Component Analysis (PCA) for gender equality and life satisfaction assessment, DT for feature selection, and predicting life satisfaction, with key determinants identifies as life expectancy, income distribution, and freedom through permutation testing and bootstrapping [12]. Khder et. al., conducts a study aiming to classify critical variables influencing life HS using ML techniques [12]. They employs supervised learning, utilizing NN training for classification and OneR models for feature selection. Their analysis reveals ‘GDP per capita’ as the primary indicator and ‘health life expectancy’ as the second most significant factor impacting life HS.

In 2020, a study on global happiness employs network learning approaches to gain deeper insights into the factors influencing happiness and their interconnections. The analysis reveals intriguing relationships, such as the lack of direct correlation between GDP per capita and generosity, and the connection between confidence in national government and freedom in life choices. Predictive modeling and Bayesian Networks are utilized to analyze historical happiness index data, with General Regression Neural Networks (GRNN) addressing predictive challenges [13]. Prashanthi et. al., conducts a study on predicting countries’ future emotional status to understand economic well-being, using the Happiness Index and SVM Kernel [16]. They introduces a supervised ML model to forecast life satisfaction scores based on parameters like environment, jobs, health, and governance. SVM performs the classification task, with a meta-ML model enhancing prediction accuracy. Another study uses ML to predict workplace happiness from office survey data, achieving 87.66% accuracy with K-Nearest Neighbors (KNN), DT, Naive Bayes, and MLP models, along with oversampling and under-sampling techniques [14]. In a study “Exploring trends in the WHR,” ‘GDP’, ‘Social Support’, and ‘Healthy Life Expectancy’ are found to be the most significant factors. Instability affects ‘social support systems’, impacting life expectancy and trade, thus affecting GDP. Multiple linear regression results in an Root Mean Squared Error (RMSE) of 0.67 and an Mean Absolute Error (MAE) of 0.50 [27]. In 2021, a study aims to analyze the world happiness dataset, extract insights, and predict HS using ML, employing algorithms like Linear Regression (LR), KNN, DT, and Random Forest(RF), with RF exhibiting the best RMSE result of 0.05, indicating its superiority in prediction accuracy [15]. On the data of 2015 to 2019, a study utilize the Happiness Index and SVM Kernel to predict countries’ future emotional status and understand their economic well-being, achieving a 56.25% accuracy rate in classification. Happiness is determined using life expectancy, experienced well-being, and ecological footprint, with the SVM effectively handling data scarcity. By combining regression models through stacking, the predictive accuracy of the model reached approximately 90% in predicting a country’s life satisfaction score [16]. Rajkumar investigates how the six cultural dimensions, as conceptualized by Hofstede and his team, relate to subjective happiness ratings across 78 countries. Data is drawn from the latest WHRs, capturing responses both pre-pandemic (2017–19) and during the pandemic (2020–21) [25]. Gunjan Anand examines the causal link between economic factors like GDP per capita, Consumer Price Index, unemployment rate, and government expenditure with the happiness index. Results suggest GDP per capita and government expenditure influence happiness, while CPI and unemployment rate do not, indicating their significance in predicting happiness [26]. The details comparison of the related literatures are in Table 1.

**Table 1:** Summary of literature review with methods, datasets and contributions.

Ref.	Year	Dataset	Methods	Best Score	Contributions
[13]	2020	World Happiness Dataset (2016-2019)	GRNN, Deep NN, RF, XGB, Ordinary Least Squares (OLS), RR, DT	GRNN (0.88)	The impact of GDP per capita on various factors such as generosity and social support, which contribute to overall happiness, is notable.
[11]	2021	World Happiness Dataset (2020)	Artificial Neural Network(ANN), SVM, Regression tree(RT)	ANN (0.83)	The World Happiness Effective Scenario’s forecast for the COVID-19 period has been observed to be rising steadily.
[12]	2022	World Happiness Dataset (2019-2020)	NN, OneR	NN (0.882)	GDP per capita is the main indicator of life happiness, followed by health life expectancy as the second most important factor.
[14]	2019	Dataset of employees of the Ministry of Public Health in Thailand	KNN, DT, Naïve Bayes, Multi-Layer Perceptron	DT (87.66)	After addressing the imbalanced data issue, the prediction accuracy surpassed that of the original data.
[17]	2022	World Happiness Dataset (2019)	Hierarchical Clustering		Three country clusters were discovered, confirming the heatmap results. Despite ranking high in generosity, the poorest cluster still falls far behind the other two groups.
[16]	2019	UN Human Development Project Dataset	Meta ML model, SVM	Meta model (90%)	Maximizing accuracy in predictions.
[15]	2021	World Happiness Dataset (2015-2021)	Linear Regression, KNN, DT, RF	RF (99.97)	Analyzing the dataset, extracting meaningful insights, and making predictions regarding HS.
[25]	2023	World Happiness Dataset (2017-2021)	Partial correlation analyses		Examining the relationship between the six dimensions of culture as defined by Hofstede and his colleagues.
[26]	2023	National Yearly Data (2010-2019)	Augmented Dickey Fuller (ADF), Johansen Co-integration Test		Explored the causal relationship between economic factors such as GDP per capita, CPI, unemployment rate, and government expenditure with the happiness index.
Ours	2024	World Happiness Dataset (2018-2023)	Stacking LRGR, Blending RGMLL	RGMLL (0.85)	Revealed important features affecting world happiness index.

Our study endeavors to predict HS utilizing a variety of ML models, including RF, Extreme Gradient Boosting (XGB), KNN, Gaussian Process (GP), Ridge Regression (RR), and Polynomial Regression (PR), DL models including MLP, Long Short-Term Memory (LSTM), Bidirectional Long Short-Term Memory (BiLSTM), and Gated Recurrent Unit (GRU), and hybrid models such as stacking ensemble learning (LRGR) and blending ensemble learning (RGMLL). Additionally, we aim to identify the factors influencing these scores through the application of XAI techniques such as SHapley Additive exPlanations (SHAP), Local Interpretable Model-Agnostic Explanations (LIME), and Eli5. Subsequent sections will introduce each model and XAI technique employed in our study individually.

## Materials and methods

In this section, we provide the details of the materials and methods of this work. First, we demonstrate the working process through an overview diagram, then describe individual elements one by one in details.

### Approach overview

**Fig 1:**
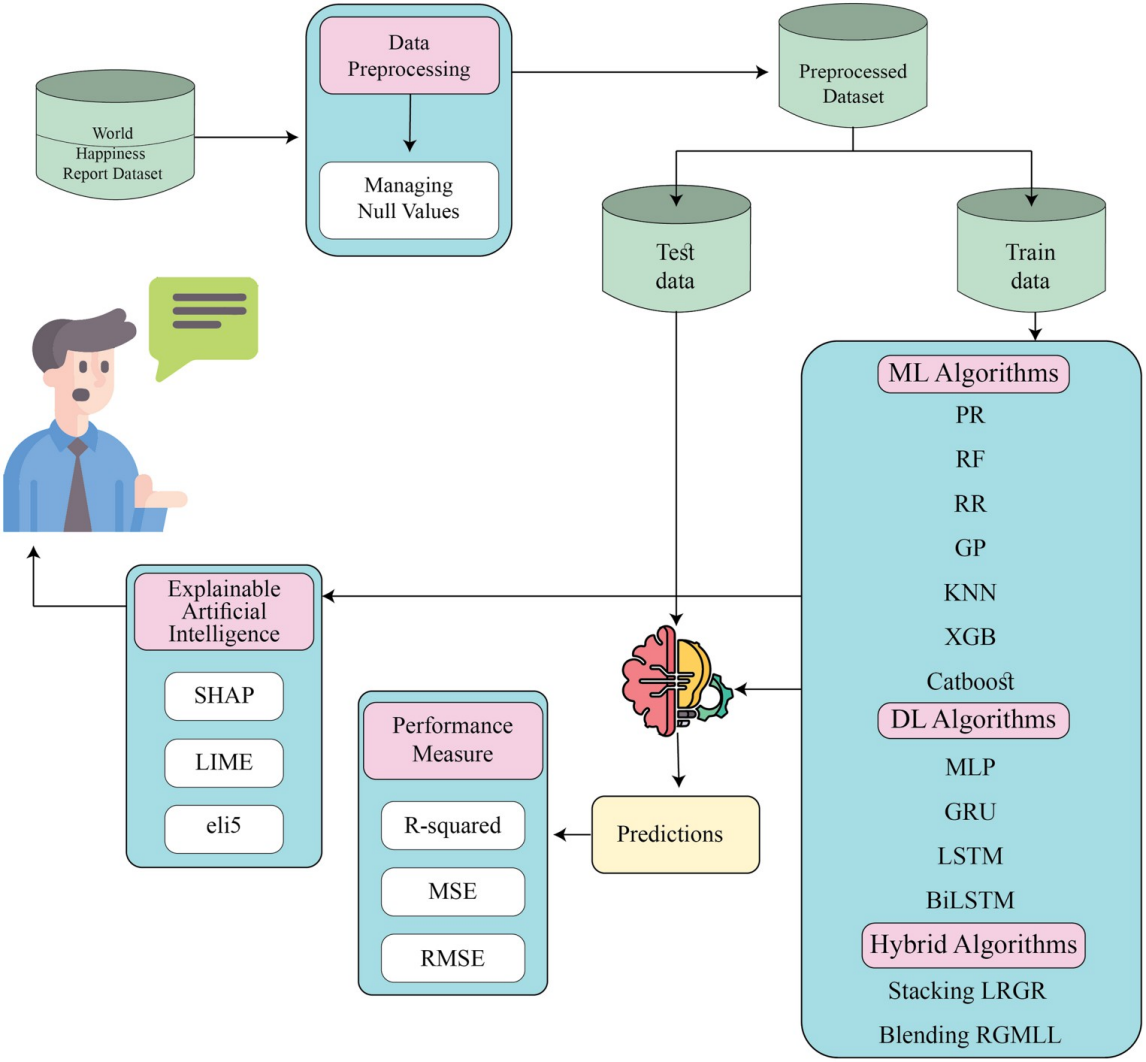
Overview of the proposed process with data processing, model development, model evaluation and explainability.

We aim to predict the HS of different countries based on the data of the WHR. This study constitutes a regression problem and we design the prediction system based on the traditional process of ML, DL, and ensemble model development. We collect data from WHR and reform the final dataset by merging the report of six consecutive years (2018-2023) and preprocess the whole dataset to make it more ML trainable. We handle the missing values and evaluate the models by dividing the dataset into 80:20, 70:30, and 50:50 training and testing partitions to check the stability of the algorithms. We train RF, XGB, KNN, GP, RR, and PR ML algorithm and MLP, LSTM, BiLSTM, and GRU DL algorithms. We also propose two ensemble model stacking LRGR and blending RGMLL and evaluate the performances of the models using R-squared (*R*^2^), MSE, and RMSE. Additionally, for more transparency and to find the hidden story of happiness and explain the black-box algorithms, we employ XAI techniques such as LIME, SHAP, and ELI5 to determine the global and local explainability. The overview of the proposed method is in Fig 1. The details of the algorithms, performance measure techniques, and XAI techniques are in the below subsections.

### Description of dataset and variables

The WHR, a pivotal annual survey initiated in 2012, provides insight into the global state of happiness, ranking countries based on their citizens perceived levels of happiness. Our analysis utilize modified online data from the report to conduct our study [10]. The data for the WHR source from the Gallup World Poll (GWP), which compiles survey responses from 156 countries. This extensive dataset tracks the evolution of key happiness indicators and their underlying factors over time. However, it’s worth noting that while the survey covers 156 countries, data availability in the earlier years is limited to only a few nations. We have compiled data from the past six years (2018-2023), encompassing all countries and divided them into three categories such as pre-pandemic (2018-19), during-pandemic (2020-21) and post-pandemic (2022-23). Table 2 provides a comprehensive description of all the features utilize for constructing the models.

**Table 2:** Description of dataset and variables.

Ref.	Category	Variable	Definition	Relationship with Happiness score	Source
[28]	Economic	GDP per capita	the total value added by resident producers plus product taxes (minus subsidies), divided by the mid-year population.	There exists a positive relationship between GDP per capita and Happiness score.	National report
[29]	National	Life expectancy	is a statistical measure indicating the average number of years a person can anticipate living, usually calculated at birth, and based on prevailing mortality rates.	There is a moderate level of positive association.	WHO
[30]	Social	Social support	It is the help or assistance individuals receive from their social networks.	social support is highly positively statistically correlated with happiness.	GWP Agency
[31]	Social	Freedom to make life choices	It’s the average level of laughter and enjoyment in waves where the happiness question wasn’t posed.	There is a positive relationship.	GWP Agency
[31]	Personal	Generosity	Generosity is the leftover after regressing the national average of donations on GDP per capita.	There is a positive relationship.	GWP Agency
[29]	National	Corruption Perception	It is the average of responses to two GWP questions: “Is corruption widespread in the government?” and “Is corruption widespread in businesses?”	There is moderate level of negative association.	GWP Agency

The target variable for prediction it the HS of countries, also known as the Life Ladder. According to the WHR, this score is derived from the average responses to the Cantril ladder life evaluation question in the GWP. Respondents are prompted to envision a ladder, where 10 represents the best possible life and 0 signifies the worst. They then rate their own current lives on this scale from 0 to 10. To effectively utilize and apply a Bayesian Network (BN) to the dataset, it needed to be discretized. The study from Beuzen provide a thorough comparison of various discretization schemes for this purpose [32].

### Data preprocessing

Missing values in features are addressed by imputing the mean of observations from other years, grouped by country for that specific feature. For instance, features like ‘healthy life expectancy’ have some missing values for the year 2023 which is substituted with the country-wise means calculated for the years 2018-2022. As a final measure, countries that have missing values for all years for a specific feature are filled using the mean calculated across all countries. After completing the aforementioned preprocessing steps, the data partitions into different ratio of training and testing datasets. The training dataset use to train the ML models, while the testing dataset is kept separate to evaluate the model’s performance on unseen data. This division ensures that the model’s performance can be accurately assess and generalize to new data.

### Descriptions of the ML algorithms

In this section, we provide the details of the ML algorithms one by one with necessary equations.

#### RF

RF is a regression technique that uses the performance of multiple DT algorithms to classify or predict the value of a variable [33–35]. That is, when RF gets a (*x*) input vector containing the values of the many evidentiary features examined for a specific training region, it constructs a number K of regression trees and averages their results. Following the growth of K such trees ${T(x)}_{1}^{K}$, the RF regression predictor is:

$$\begin{array}{c} {\hat{f}}_{\text{rf}}^{K}\left(x\right)=\frac{1}{K}\sum_{k=1}^{K}T\left(x\right) \end{array}$$

By allowing the trees to develop from several training data subsets produced by a process known as bagging, RF promotes the variety of the trees and prevents the correlation between the various trees. More stability is thereby attained, increasing prediction accuracy while strengthening the system’s resistance to even small changes in the input data [33].

#### XGB

Recently, XGB, a ML algorithm, was invented and is now widely employed across multiple fields. Numerous applications will benefit from this method’s organization, portability, and flexibility [36]. By integrating Cause Based DT (CBDT) and Gradient Boosting Machine (GBM) into a single, efficient method, this strategy improved the tree-boosting approach’s capacity to handle almost all data kinds rapidly and reliably. Additionally, this method provides effective and efficient solutions to new optimization issues, particularly when accuracy and efficiency trade-offs are taken into account [37].

#### KNN

A non-parametric technique known as KNN regression uses the average of observations within the same neighborhood to roughly approximate the relationship between independent variables and the continuous outcome [38]. The neighborhood in which KNN regression is applied is determined by measuring the distance between the data points; the most popular distance measure is Euclidean Distance [39], which is given for two data points (*x*_*i*_ and *x*_*j*_) in a d-dimensional space by:

$$\begin{array}{c} d\left(x_{i},x_{j}\right)=\sqrt{\sum_{k=1}^{d}\left(x_{i},x_{j}\right)} \end{array}$$

where, *x*_*i*_*k* and *x*_*j*_*k* are the kth features of points *x*_*i*_ and *x*_*j*_ respectively. *d* is the number of dimensions (features) in the dataset.

#### GP

One kind of probabilistic and non-parametric approach is predicated on the idea that the function that needs to be learned comes from a GP [40]. The model can produce predictions with a well-defined uncertainty thanks to this assumption, which is helpful for tasks like active learning and uncertainty-aware decision-making. In terms of math,

$$\begin{array}{c} f\left(x\right)=GP(m\left(x\right),k\left(x,x^{\prime }\right)) \end{array}$$

where, *m*(*x*) is the mean function. *k*(*x*, *x*′) is the covariance function, which determines the correlation between points *x* and *x*′.

#### RR

RR is a basic regularisation method where the OLS objective function is modified by adding a penalty term to address multicollinearity problems and regularises the model [41]. In ridge regression, the objective function is provided by:

$$\begin{array}{c} min_{\beta }∥Y-X\beta {∥}_{2}^{2}+\gamma ∥\beta {∥}_{2}^{2} \end{array}$$

where, *Y* is the vector of observed target values. *X* is the matrix of input features. *β* is the vector of regression coefficients to be estimated. *γ* is the regularization parameter. $∥.{∥}_{2}^{2}$ represents the squared L2 norm. Finding the values of the coefficient vector *β* that minimize the sum of the RSS and the regularisation penalty is the aim of RR.

#### PR

An n-degree polynomial is used to represent the relationship between the independent variable (*x*) and the dependent variable (*y*) in PR, a type of LR [42]. With a polynomial of degree n, the PR equation is as follows:

$$\begin{array}{c} y\left(x\right)=\beta_{0}+\beta_{1}x+\beta_{2}x^{2}+\ldots +\beta_{n}x^{n}+\epsilon \end{array}$$ (5)

where, *y* is the dependent variable (target). *x* is the independent variable (feature). *β*_0_, *β*_1_, *β*_2_, …, *β*_*n*_ are the coefficients of the polynomial terms. *ϵ* is the error term. Finding the coefficients *β*_0_, *β*_1_, *β*_2_, …, *β*_*n*_ that most closely match the observed data points is the aim of PR.

### Descriptions of the DL algorithms

**MLP:** MLP is a type of artificial neural network composed of an input layer, one or more hidden layers, and an output layer [43]. Each layer contains neurons that compute outputs using weights *W*, biases *b*, and activation functions *ϕ*. For an input vector *x*, the output of each neuron in a layer is calculated as:

$$\begin{array}{c} z=W\cdot x+b \end{array}$$

$$\begin{array}{c} a=\phi (z) \end{array}$$

where *W* is the weight matrix, *b* is the bias vector, and *a* is the activation. This process is repeated for each layer, passing the activations to the subsequent layer until the final output layer. The MLP uses backpropagation for training, adjusting weights and biases to minimize the error between predicted and actual outputs [44].

#### LSTM

LSTM is a type of recurrent neural network (RNN) designed to handle sequences of data and long-term dependencies [45]. It consists of memory cells, each with a cell state *c*_*t*_ and hidden state *h*_*t*_. The LSTM cell uses input (*i*_*t*_), forget (*f*_*t*_), and output (*o*_*t*_) gates, along with a candidate cell state ($\tilde{c_{t}}$). The equations governing these components are:

$$\begin{array}{c} i_{t}=\sigma \left(W_{i}\cdot \left[h_{t-1},x_{t}\right]+b_{i}\right) \end{array}$$

$$\begin{array}{c} f_{t}=\sigma \left(W_{f}\cdot \left[h_{t-1},x_{t}\right]+b_{f}\right) \end{array}$$

$$\begin{array}{c} o_{t}=\sigma \left(W_{o}\cdot \left[h_{t-1},x_{t}\right]+b_{o}\right) \end{array}$$

$$\begin{array}{c} \tilde{c_{t}}=\text{tanh}\left(W_{c}\cdot \left[h_{t-1},x_{t}\right]+b_{c}\right) \end{array}$$

$$\begin{array}{c} c_{t}=f_{t}*c_{t-1}+i_{t}*\tilde{c_{t}} \end{array}$$

$$\begin{array}{c} h_{t}=o_{t}*\text{tanh}\left(c_{t}\right) \end{array}$$

where *σ* is the sigmoid function, *W* and *b* are weights and biases, respectively, and *x*_*t*_ is the input at time step *t*. These gates regulate the flow of information, allowing LSTMs to capture long-term dependencies and mitigate the vanishing gradient problem [46].

#### BiLSTM

BiLSTM network is an extension of the LSTM network that processes data in both forward and backward directions to capture context from both past and future states. Each BiLSTM cell consists of two LSTM layers: one that processes the input sequence forward $\vec{h_{t}}=o_{t}*\text{tanh}\left(\vec{c_{t}}\right)$ and another that processes it backward $\overset{\leftarrow }{h_{t}}=o_{t}*\text{tanh}\left(\overset{\leftarrow }{c_{t}}\right)$. The final output at each time step *t* is the concatenation of $\vec{h_{t}}$ and $\overset{\leftarrow }{h_{t}}$:

$$\begin{array}{c} h_{t}=\left[\vec{h_{t}},\overset{\leftarrow }{h_{t}}\right] \end{array}$$

This structure allows BiLSTMs to leverage context from both directions, improving performance on tasks where understanding the full sequence is crucial [47].

#### GRU

GRU network is a type of RNN that efficiently captures dependencies in sequence data using gating mechanisms. Unlike LSTM, GRU has fewer gates, making it simpler and computationally less expensive. Each GRU cell has a reset gate *r*_*t*_ and an update gate *z*_*t*_, which control the flow of information. The equations governing these components are:

$$\begin{array}{c} z_{t}=\sigma \left(W_{z}\cdot \left[h_{t-1},x_{t}\right]+b_{z}\right) \end{array}$$

$$\begin{array}{c} r_{t}=\sigma \left(W_{r}\cdot \left[h_{t-1},x_{t}\right]+b_{r}\right) \end{array}$$

$$\begin{array}{c} \tilde{h_{t}}=\text{tanh}\left(W_{h}\cdot \left[r_{t}*h_{t-1},x_{t}\right]+b_{h}\right) \end{array}$$

$$\begin{array}{c} h_{t}=\left(1-z_{t}\right)*h_{t-1}+z_{t}*\tilde{h_{t}} \end{array}$$

where *σ* is the sigmoid function, tanh is the hyperbolic tangent function, *W* and *b* are weights and biases, respectively, and *x*_*t*_ is the input at time step *t*. The update gate *z*_*t*_ determines how much of the previous state *h*_*t*−1_ to keep, while the reset gate *r*_*t*_ controls how much of the previous state to forget when computing the candidate activation $\tilde{h_{t}}$. This gating mechanism allows GRUs to efficiently manage long-term dependencies and mitigate the vanishing gradient problem [48].

### Descriptions of the ensemble models

#### Stacking LRGR

**Fig 2:**
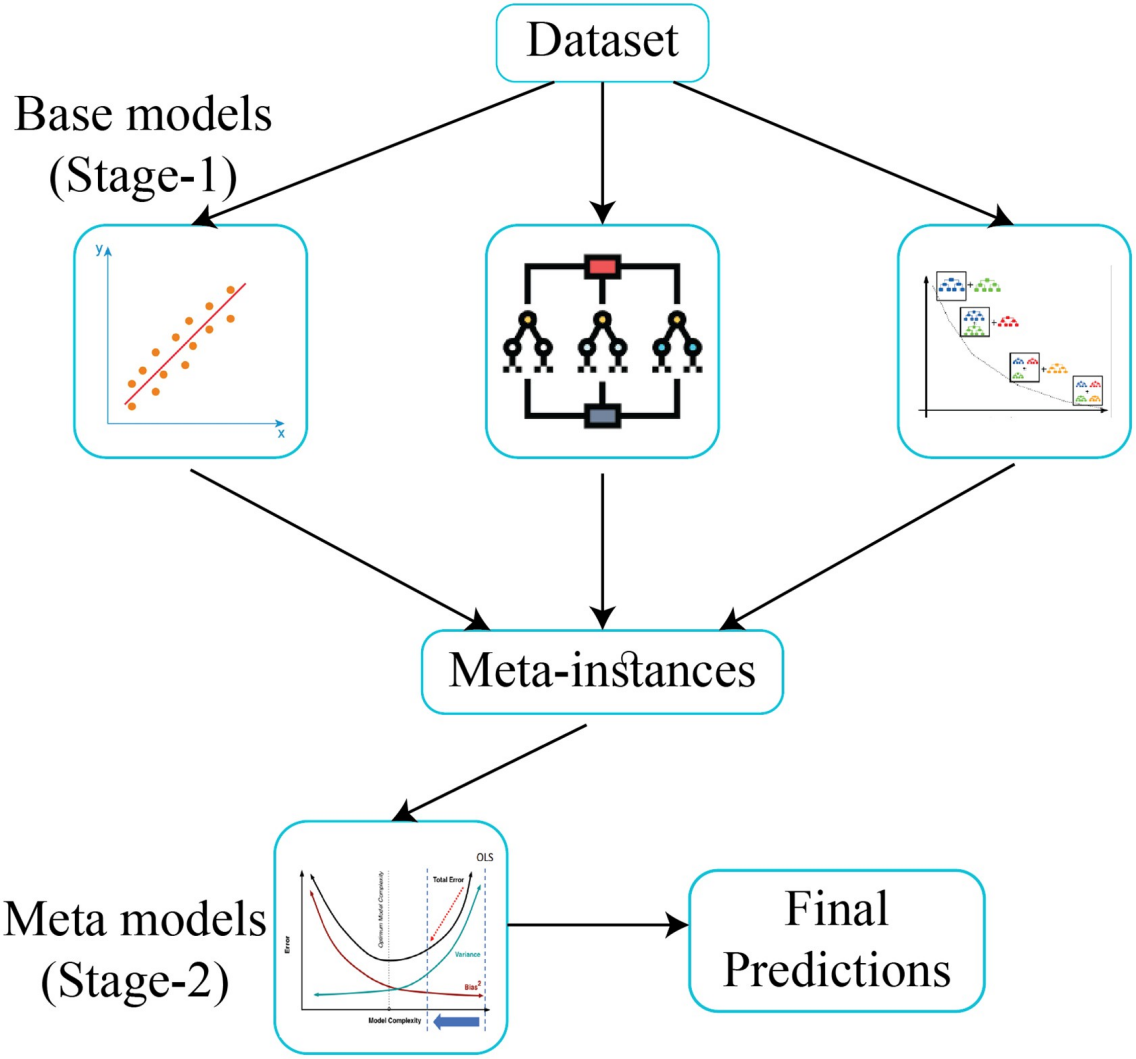
Block diagram of proposed LRGR stacking ensemble learning model.

Stacking ensemble learning leverages the complementary strengths of various base models to boost performance and generalization [49]. It involves two phases: training base models and training a meta-model [50]. First, the original data is split into a training set and a testing set. The training set undergoes k-fold cross-validation, where it’s divided into k parts, and each part is trained using the remaining k-1 parts, generating predictions. These predictions form a new training set for the meta-model. In the second phase, the predictions from the base models’ testing sets are combined to create the meta-model’s testing set. The meta-model is then trained on this new dataset. In stacking ensemble learning, selecting appropriate base and meta models is essential. In this study, three types of base models are used: a general regression model (LR), a tree-based model (RF), and a boosting model (GB). A regularization technique (RR), is chosen as the meta model. The workflow is given in Fig 2.

The process begins with training the base learners on the given dataset $D={\left\{\left(x_{i},y_{i}\right)\right\}}_{i=1}^{n}$, where $x_{i}\in R^{d}$ and $y_{i}\in R$. Each base learner generates its predictions:

$$\begin{array}{c} {\hat{y}}_{\text{LR},i}=x_{i}^{⊤}\beta \end{array}$$

$$\begin{array}{c} {\hat{y}}_{\text{RF},i}=\text{RF}\left(x_{i}\right) \end{array}$$

$$\begin{array}{c} {\hat{y}}_{\text{GB},i}=\text{GB}\left(x_{i}\right) \end{array}$$

These predictions are then used to construct a new meta-training dataset $D^{\prime }$, where each instance ${\hat{y}}_{i}$ is a vector of the base learners’ predictions:

$$\begin{array}{c} D^{\prime }={\left\{({\hat{y}}_{i},y_{i})\right\}}_{i=1}^{n}\;\text{with}\;{\hat{y}}_{i}={\left({\hat{y}}_{\text{LR},i},{\hat{y}}_{\text{RF},i},{\hat{y}}_{\text{GB},i}\right)}^{⊤} \end{array}$$

The meta learner, RR, is trained on this new dataset. The RR model predicts the final output ${\hat{y}}_{i}$ using:

$$\begin{array}{c} {\hat{y}}_{i}=\theta^{⊤}{\hat{y}}_{i} \end{array}$$

The parameters ***θ*** are optimized by minimizing the regularized loss function:

$$\begin{array}{c} \underset{\theta }{\text{min}}\sum_{i=1}^{n}{\left(y_{i}-\theta^{⊤}{\hat{y}}_{i}\right)}^{2}+\lambda {∥\theta ∥}^{2} \end{array}$$

where λ is the regularization parameter. For a new input x^*^, the final prediction ${\hat{y}}_{\text{final}}$ is computed by combining the base learners’ predictions through the trained meta learner:

$$\begin{array}{c} {\hat{y}}_{\text{final}}=\theta^{⊤}{\left({\hat{y}}_{\text{LR}}\left(x^{*}\right),{\hat{y}}_{\text{RF}}\left(x^{*}\right),{\hat{y}}_{\text{GB}}\left(x^{*}\right)\right)}^{⊤} \end{array}$$

This combination of base learners’ outputs through a meta learner harnesses the strengths of individual models, leading to improved predictive performance.

**Algorithm 1** Stacking LRGR

1: **Input:** Train dataset $D={\left\{(x_{i},y_{i})\right\}}_{i=1}^{n}$ where $x_{i}\in R^{n},y_{i}\in Y$

2: **Output:** An ensemble regressor *R*

3: **Step 1: Learn base regressors**

4: **for** *k* ← 1 to *K* **do**

5:  Learn a base regressor *r*_*k*_ based on D

6: **end for**

7: **Step 2: Construct new datasets**

8: **for** *i* ← 1 to *n* **do**

9:  Construct a new data set $\left\{x_{i}^{\prime },y_{i}\right\}$ where $x_{i}^{\prime }=\{r_{1}(x_{i}),r_{2}(x_{i}),\ldots ,r_{K}(x_{i})\}$

10: **end for**

11: **Step 3: Learn a meta regressor**

12: Learn a new regressor *r*′ on $\left\{x_{i}^{\prime },y_{i}\right\}$

13: **Return:** *R*(**x**) = *r*′(*r*_1_(**x**), *r*_2_(**x**), …, *r*_*K*_(**x**)).

#### Blending RGMLL

**Fig 3:**
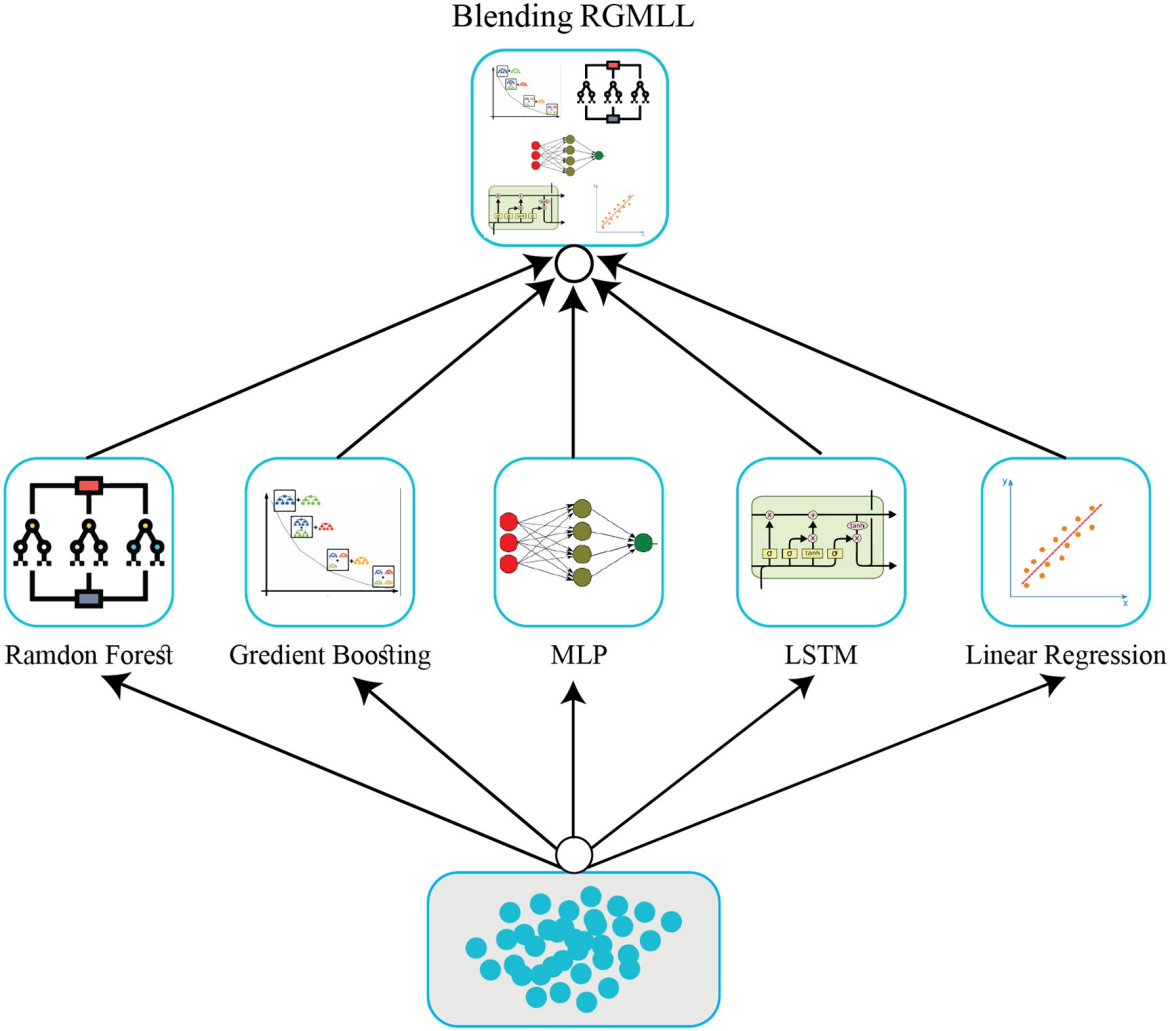
Block diagram of proposed RGMLL blending ensemble learning model.

In this study, we develop the RGMLL regression model, a blending ensemble learning approach combining five algorithms: RF, GBM, MLP, LSTM, and LR. Blending, unlike stacking, uses a small validation set (10–15% of the training data) instead of out-of-fold predictions for training the meta model. This ensemble method integrates the strengths of its individual models, enhancing predictive accuracy and performance. Ensembles also reduce prediction variability, improving reliability and overall predictive capabilities. The workflow is given in Fig 3. Our proposed ensemble model incorporates

- Two ML models: RF and GBM
- Two DL models: MLP and LSTM
- One general regression model: LR

Initially, we train the base models on the training dataset $D={\left\{\left(x_{i},y_{i}\right)\right\}}_{i=1}^{n}$, where $x_{i}\in R^{d}$ and $y_{i}\in R$. Each base model generates its respective predictions:

$$\begin{array}{c} {\hat{y}}_{\text{RF},i}=\text{RF}\left(x_{i}\right) \end{array}$$

$$\begin{array}{c} {\hat{y}}_{\text{GB},i}=\text{GB}\left(x_{i}\right) \end{array}$$

$$\begin{array}{c} {\hat{y}}_{\text{MLP},i}=\text{MLP}\left(x_{i}\right) \end{array}$$

$$\begin{array}{c} {\hat{y}}_{\text{LSTM},i}=\text{LSTM}\left(x_{i}\right) \end{array}$$

To train the meta model, we construct a validation dataset $V={\left\{\left(x_{j},y_{j}\right)\right\}}_{j=1}^{m}$. The base models generate predictions on this validation dataset to create a new dataset $D^{\prime }$ for the meta model. Each instance ${\hat{y}}_{j}$ in this new dataset is a vector of the predictions from the base models:

$$\begin{array}{c} D^{\prime }={\left\{({\hat{y}}_{j},y_{j})\right\}}_{j=1}^{m}\;\text{where}\;{\hat{y}}_{j}={\left({\hat{y}}_{\text{RF},j},{\hat{y}}_{\text{GB},j},{\hat{y}}_{\text{MLP},j},{\hat{y}}_{\text{LSTM},j}\right)}^{⊤} \end{array}$$

We then train the meta model, LR, on this newly constructed dataset to predict the final output ${\hat{y}}_{j}$:

$$\begin{array}{c} {\hat{y}}_{j}=\beta^{⊤}{\hat{y}}_{j} \end{array}$$

The coefficients ***β*** of the LR model are optimized by minimizing the MSE:

$$\begin{array}{c} \underset{\beta }{\text{min}}\sum_{j=1}^{m}{\left(y_{j}-\beta^{⊤}{\hat{y}}_{j}\right)}^{2} \end{array}$$

For a new input x^*^, the final prediction ${\hat{y}}_{\text{final}}$ is obtained by aggregating the predictions from the base models through the trained LR model:

$$\begin{array}{c} {\hat{y}}_{\text{final}}=\beta^{⊤}{\left({\hat{y}}_{\text{RF}}\left(x^{*}\right),{\hat{y}}_{\text{GB}}\left(x^{*}\right),{\hat{y}}_{\text{MLP}}\left(x^{*}\right),{\hat{y}}_{\text{LSTM}}\left(x^{*}\right)\right)}^{⊤} \end{array}$$

This blending method effectively leverages the strengths of the diverse base models, enhancing predictive performance through the integration of their outputs by the LR meta model.

**Algorithm 2** Blending RGMLL

1: **Input:** Train dataset $D={\left\{(x_{i},y_{i})\right\}}_{i=1}^{n}$ where $x_{i}\in R^{n},y_{i}\in Y$, validation dataset $V={\left\{(x_{j},y_{j})\right\}}_{j=1}^{m}$

2: **Output:** An ensemble regressor *R*

3: **Step 1: Learning base regressors**

4: **for** *k* ← 1 to *K* **do**

5:  Learn a base regressor *r*_*k*_ based on D

6: **end for**

7: **Step 2: Predict on validation set**

8: **for** *j* ← 1 to *m* **do**

9:  Construct a new validation data set $\left\{x_{j}^{\prime },y_{j}\right\}$ where $x_{j}^{\prime }=\{r_{1}(x_{j}),r_{2}(x_{j}),\ldots ,r_{K}(x_{j})\}$

10: **end for**

11: **Step 3: Learn a meta regressor**

12: Learn a new regressor *r*′ based on the newly constructed validation data set

13: **Step 4: Combine predictions**

14: For a new instance **x**, obtain predictions from base regressors {*r*_1_(**x**), *r*_2_(**x**), …, *r*_*K*_(**x**)}

15: Predict the final output using the meta regressor: *R*(**x**) = *r*′(*r*_1_(**x**), *r*_2_(**x**), …, *r*_*K*_(**x**))

16: **Return:** *R*

### Performance measure metrics

Here, *y*_*i*_ represents the predicted value, and the *x*_*i*_ element is the observed value. The regression method predicts the *y*_*i*_ element for the corresponding *x*_*i*_ element of the dataset.

**Coefficient of Determination (*R*^2^).** The coefficient of determination [51] can be interpreted as the proportion of the variance in the dependent variable that is predictable from the independent variables. Which can range from 0 to 1.

$$\begin{array}{c} R^{2}=1-\frac{\sum_{i=1}^{n}{\left(y_{i}-x_{i}\right)}^{2}}{\sum_{i=1}^{n}{\left(\bar{x}-x_{i}\right)}^{2}} \end{array}$$

**MSE:** It is defined as Mean or Average of the square of the difference between actual and estimated values [52, 53].

$$\begin{array}{c} \text{MSE}=\frac{1}{n}\sum_{i=1}^{n}{\left(y_{i}-{\hat{x}}_{i}\right)}^{2} \end{array}$$

**RMSE.** The underlying assumption when presenting the RMSE is that the errors are unbiased and follow a normal distribution.

$$\begin{array}{c} \text{RMSE}=\sqrt{\frac{1}{n}\sum_{i=1}^{n}{\left(y_{i}-{\hat{x}}_{i}\right)}^{2}} \end{array}$$

### Descriptions of the explainable AI techniques

#### LIME

Local surrogate models that explain black box ML model predictions are called “LIME” [54]. Why the black box model predict a data instance explain by training local surrogate models instead of global ones. LIME changes samples and predicts black box models. This dataset trains an interpretable model like Lasso or a DT using proximity-based weights. Instead of a strong global approximation, local surrogate models approximate the black box model’s instance predictions. In mathematical terms, local surrogate models, while adhering to the requirement of interpretability, can be represented as follows:

$$\begin{array}{c} \underset{g\in G}{\text{argmin}}L\left(f,g,\pi_{x}\right)+\Omega \left(g\right) \end{array}$$

A loss-minimizing model *g*, like LR *L* explains example *x*. As with XGB, this loss evaluates how well the explanation matches model *f* predictions. *G*, with a simple model *Ω*(*g*) and fewer characteristics, includes all plausible explanations, including LR. The proximity measure *π*_*x*_ defines the size of the neighborhood surrounding instance *x*. LIME lets users set complexity, like the LR model’s maximum features, to optimize loss. LIME supports tabular, text, and image data, unlike other techniques. Inconsistent LIME replies are troublesome. Two nearby data points may have distinct meanings, according to simulations [55]. LIME explanations can be altered by data scientists to hide biases [56].

#### SHAP

Lundberg and Lee (2017) describe individual predictions using SHAP [57]. It employs optimum game-theoretic Shapley values. The explanation approach calculates coalitional game theory Shapley values for each feature’s prediction contribution. These values divide prediction evenly among feature values as coalition members. LIME and Shapley values links via a novel linear model-like additive feature attribution mechanism. It describes both methods and unites interpretable ML. The SHAP explains:

$$\begin{array}{c} g\left(z^{\prime }\right)=\phi_{0}+\sum_{j=1}^{M}\phi_{j}z_{j}^{\prime } \end{array}$$

SHAP explanation model *g* uses coalition vectors *z*′(*binary*0, 1) for feature presence and Shapley values *ϕ*_*j*_ for feature attributions. The author of Interpretable ML thinks “SHAP” was named because it collects feature superpixels instead of pixels in picture data [58]. Coalition vector *z*′s describe “present” (1) or “absent” (0) attributes, like Shapley values, which compute contributions by “playing” or “not playing” feature values. *ϕ*’s may be explained using linear coalition models. The coalition vector *x*6′ for interest *x* has all 1’s, indicating all feature values are “present”. Write the simplified formula:

$$\begin{array}{c} g\left(x^{\prime }\right)=\phi_{0}+\sum_{j=1}^{M}\phi_{j} \end{array}$$

Interpretable ML’s author attributes SHAP’s popularity to its quick tree-based model implementation. Slow computation is the largest Shapley value adoption barrier. SHAP may be used to purposefully falsify data and hide biases, according to Slack et al. [56].

#### Eli5

The Eli5 package aims to debug and explain ML classifiers, supporting Scikit-learn algorithms. It clarifies feature weights, and predictions, displays DTs, shows feature importance, and explains predictions made by DTs and tree-based ensembles [59]. With functions like ‘show_weights()’ for global model interpretation, providing explanations for classifier parameters, and ‘show_prediction()’ for local interpretation, explaining classifier predictions. Global interpretation helps comprehend a model’s logic across all potential outcomes [60]. These methods have broader applications on a population scale, addressing issues like drug consumption trends, climate change, and the global public health challenge of suicide [61]. Global interpretation techniques were employed to assess the significance of each feature in algorithm outputs. For local interpretation, the focus was on explaining the rationale behind individual predictions [60]. In sentiment analysis exploration, text samples were scrutinized to uncover why the model categorized instances as positive or negative, with each word carrying a weight impacting classification. Leveraging the Eli5 library enabled pinpointing specific features influencing the classification of individual samples.

## Results and discussion

In this section, we present all the obtained results according to the list of contributions. We first show the explanatory data analysis and descriptive statistics in details and then show the performances of the ML, DL and proposed ensemble models. Finally, we present the explainability result to revel the hidden story and the impact of COVID-19 on the happiness.

### Explanatory data analysis with descriptive statistics

The following Table 3 presents descriptive statistics of the features used in this study which contains mean, variance, standard deviation, minimum, maximum and quartiles (25th, 50th, 75th percentiles) for each feature. The mean of the feature freedom to make life choices is 0.62, with a variance of approximately 0.05, indicating moderate variability around the mean. The distribution of this feature appears relatively symmetric, with the median of it also being 0.63. The range spans from 0.00 to 0.97, with the majority of individuals falling between 0.47 and 0.80. The feature GDP per capita emerged as positively skewed as the mean (5.14) greater than the median (1.95) with standard deviation 4.26 which indicating large variability in the range of 0.00 to 11.66.

**Table 3:** Descriptive statistics of the variables for better understanding the nature of the dataset.

index	variance	mean	std	min	25%	50%	75%	max
Freedom to make life choices	0.05	0.62	0.22	0.00	0.47	0.63	0.80	0.97
GDP per capita	18.15	5.14	4.26	0.00	1.05	1.95	9.50	11.66
Generosity	0.02	0.09	0.15	-0.30	-0.02	0.10	0.19	0.60
Happiness score	1.23	5.48	1.11	1.86	4.64	5.51	6.26	7.84
Healthy life expectancy	1049.76	32.00	32.40	0.00	0.66	1.04	66.10	77.28
Perceptions of corruption	0.12	0.42	0.34	0.00	0.09	0.34	0.78	0.94
Social support	0.08	0.96	0.29	0.00	0.79	0.91	1.17	1.64

**Fig 4:**
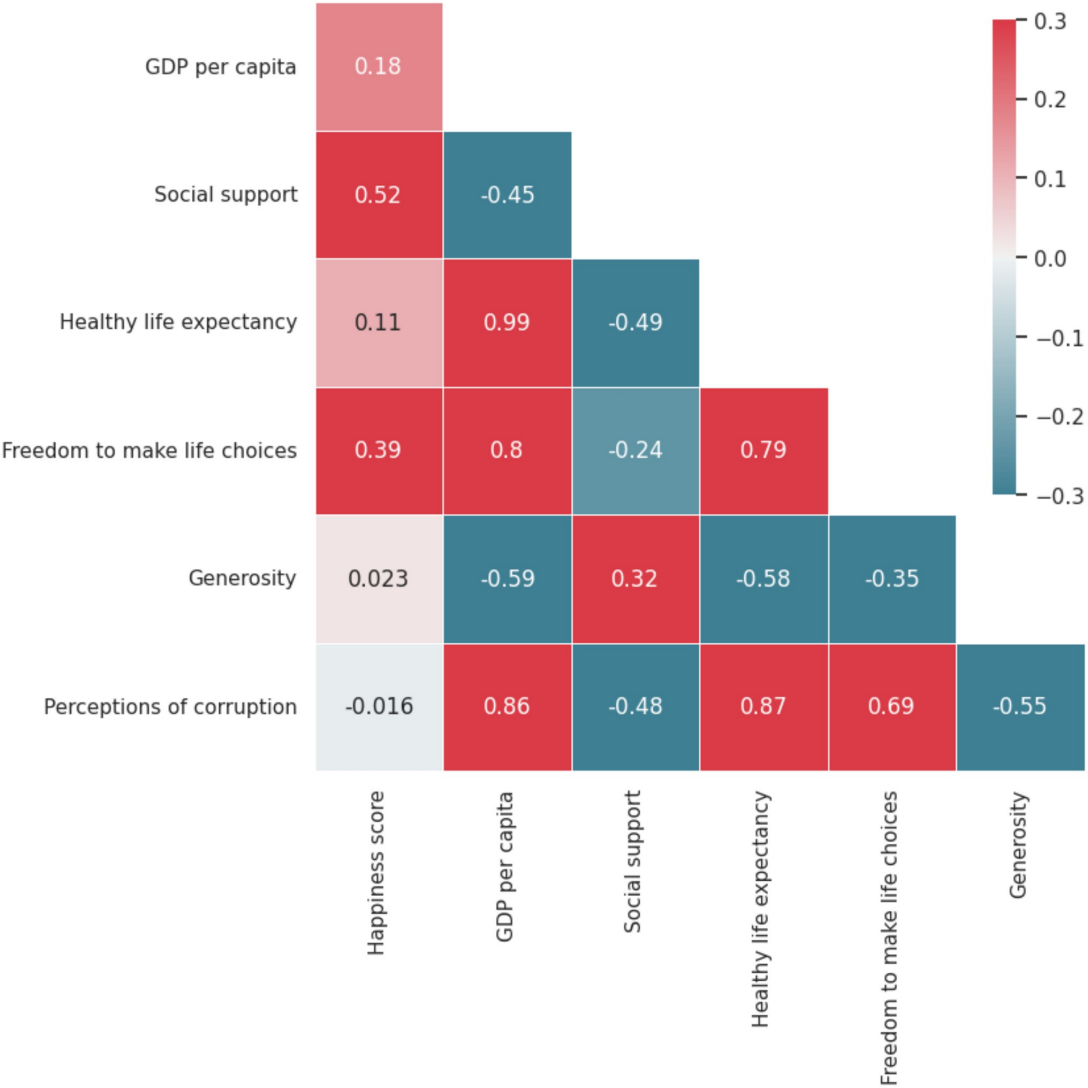
Correlation heatmap representing the strength of relationship among the features.

Similarly, Generosity, Happiness score and Social support showing relatively symmetric distribution as their mean and median are approximately same with small variability. Other features, Healthy life expectancy and Perceptions of corruption revealing some kind of positive skewness as their mean are greater than median. Here is the correlation heatmap of the features in Fig 4 showing the Pearson’s correlation coefficient among the features.

As the heatmap there is highest correlation between ‘Healty life expectancy’ and ‘GDP per capita’ (0.99). GDP per capita also have some greater level of correlation with Freedom to make life choices and Perception of corruption. Healty life expectancy also moderately correlated with Freedom to make life choices and Perception of corruption. Freedom to make life choices and Perception of corruption are moderately related.

### Results of hyperparameter tuning

Hyperparameters in ML refers to a parameter whose value is set before the learning process begins. These parameters influence the learning process itself, rather than being learned from the training data. The importance of hyperparameters lies in their ability to significantly impact the performance of ML models. By tuning hyperparameters appropriately, it is possible to improve the model’s ability to generalize to unseen data, thus enhancing its predictive accuracy and efficiency. We perform GreadSearchCV process to find the suitable hyperparameters for the algorithms then apply dataset for training. The hyperparameters of individual model with suitable values are in Table 4.

**Table 4:** Hyperparameters of the models with suitable values.

Model	Attributes and Model Parameter
RR	n_estimators = 100
XGB	verbosity = 0
KNN	n_neighbors = 12
RR	alpha = 0.01
GP	kernel = kernel, n_restarts_optimizer = 10
PR	degree = 2, include_bias = False

### Performance of ML and stacking LRGR models

**Fig 5:**
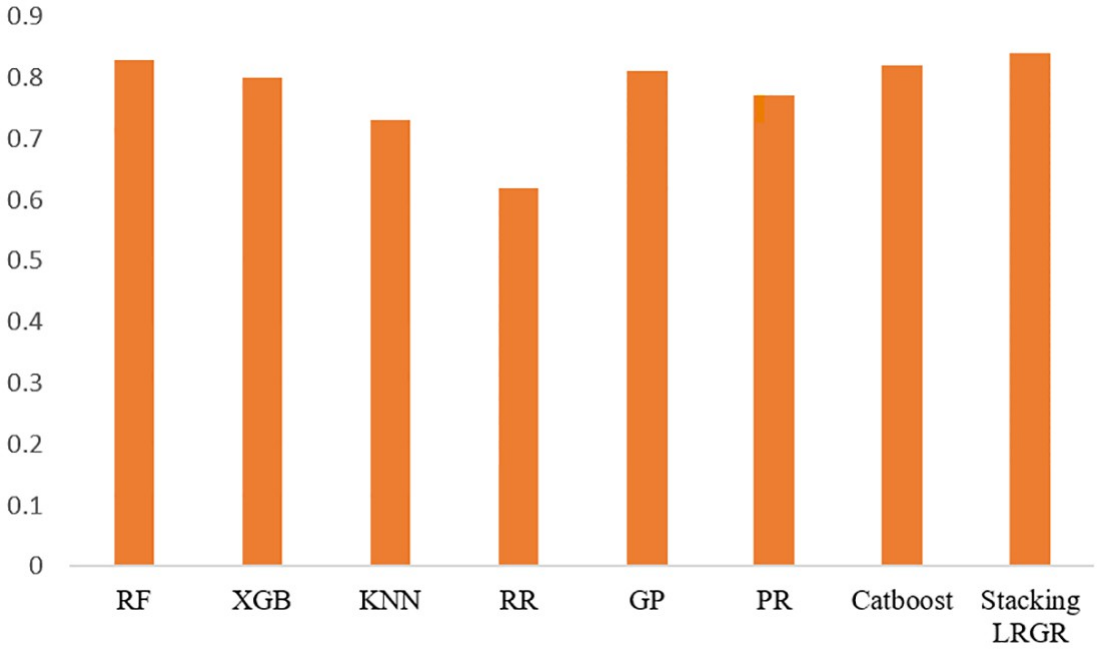
Model performance(*R*^2^) for the ML and stacking LRGR models.

We employ various ML models to predict the HS of the six years data. We train different ML algorithms on the (80%, 70%, and 50%) training data on same experimental setup and assess the performances of each models by different ratio of the testing data. We summarizes the performance of both standard and the best-performing models using an 80:20 ratio that are in Table 5. The analysis reveals that RF exhibit superior performance compare to other single models, achieving an *R*^2^ of 0.83, MSE of 0.19, and RMSE of 0.44. Following closely, GP demonstrated notable performance with an *R*^2^ of 0.81, MSE of 0.20, and RMSE of 0.45. Additionally, XGB delivered satisfactory results with an R2 of 0.80, MSE of 0.22, and RMSE of 0.47 when predicting HS on the test data. But the Stacking LRGR model outperformed all the single models with *R*^2^ of 0.84, MSE of 0.18, and RMSE of 0.43. This suggests that the combined synergy of multiple models can surpass the performance of any single model. The *R*^2^ values of the models are also presented in the Fig 5 below. Where *Y* axis representing the *R*^2^ value and *X* axis representing the different models.

**Table 5:** ML models performance with 80:20 training vs testing ratio.

Model	*R* ^2^	MSE	RMSE
RF	0.83	0.19	0.44
XGB	0.80	0.22	0.47
KNN	0.73	0.30	0.55
RR	0.62	0.42	0.65
GP	0.81	0.20	0.45
PR	0.77	0.25	0.50
Stacking LRGR	0.84	0.18	0.43

The table above summarizes the outputs of the ML models when applied to a training and test ratio of 70:30 (Table 6). The analysis revealed that RF exhibited superior performance compared to other models, achieving an *R*^2^ of 0.82, MSE of 0.21, and RMSE of 0.46. Following closely, XGB demonstrated notable performance with an R2 of 0.81, MSE of 0.21, and RMSE of 0.47. Additionally, GP delivered satisfactory results with an R2 of 0.80, MSE of 0.23, and RMSE of 0.47 when predicting HS on the test data.

**Table 6:** Model performance with 70:30 training vs testing ratio.

Model	*R* ^2^	MSE	RMSE
RF	0.82	0.21	0.46
XGB	0.81	0.21	0.47
KNN	0.71	0.34	0.58
RR	0.64	0.42	0.65
GP	0.80	0.23	0.47
PR	0.78	0.25	0.50

When applying the ML model to the dataset divided into a train-test ratio of 50:50, RF displayed superior performance compared to other models, achieving an *R*^2^ of 0.78, MSE of 0.25, and RMSE of 0.50. Following closely, PR demonstrated notable performance with an R2 of 0.77, MSE of 0.26, and RMSE of 0.51. Additionally, XGB delivered satisfactory results with an *R*^2^ of 0.76, MSE of 0.27, and RMSE of 0.52 when predicting HS on the test data. Displayed in the Table 7.

**Table 7:** Model performance with 50:50 training vs testing ratio.

Model	*R* ^2^	MSE	RMSE
RF	0.78	0.25	0.50
XGB	0.76	0.27	0.52
KNN	0.69	0.34	0.58
RR	0.63	0.41	0.64
GP	0.73	0.29	0.54
PR	0.77	0.26	0.51

### Performance of the DL and blending RGMLL models

**Fig 6:**
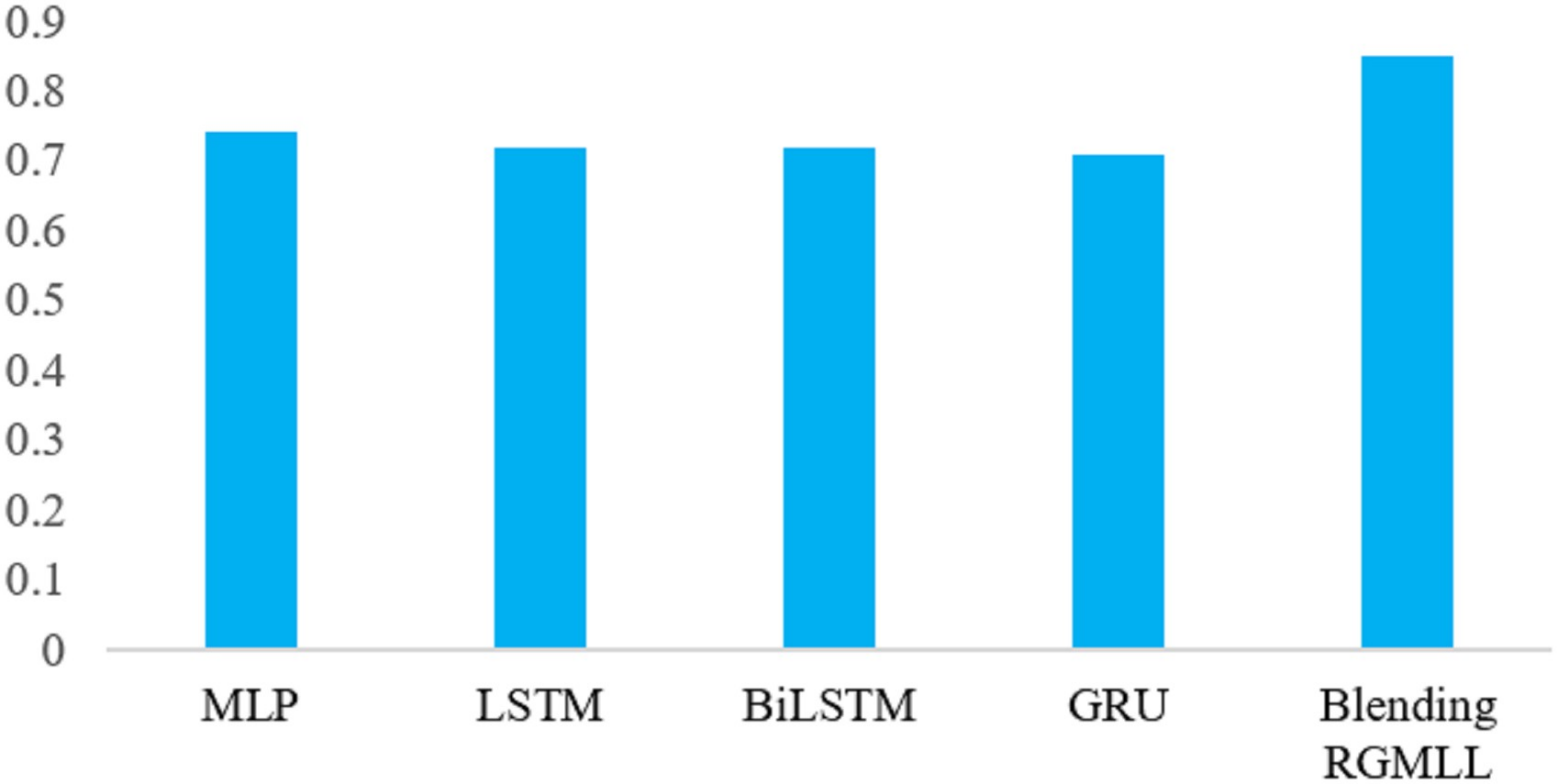
Model performance(*R*^2^) for the DL models.

The performance of various DL models and the blending RGMLL model is shown in below Table 8. Which shows that the predictive performance of the blending RGMLL model is superior compared to the other models used in this analysis with *R*^2^ of 0.85, MSE of 0.15, and RMSE of 0.38 following by MLP, LSTM and BiLSTM respectively. Where LSTM and BiLSTM performed almost similarly. MLP performs better than other single DL models with *R*^2^ of 0.74, MSE of 0.23, and RMSE of 0.48. And GRU is the worst performer among the DL models when predicting HS on the test data. Which is also represents by the Fig 6.

**Table 8:** Performance of the DL models.

Model	*R* ^2^	MSE	RMSE
MLP	0.74	0.23	0.48
LSTM	0.72	0.25	0.50
BiLSTM	0.72	0.25	0.49
GRU	0.71	0.25	0.50
Blending RGMLL	0.85	0.15	0.38

The *R*^2^ values of the models are also presented in the Fig 6 above. Where *Y* axis representing the *R*^2^ value and *X* axis representing the different models.

### Result of explainability

In this section, we show the result of the XAI techniques to analyze the predictions made by the optimal algorithm across three distinct time categories: pre-COVID, during-COVID, and post-COVID. The aim is to identify and understand the factors that influence happiness during these different temporal periods as well as the impact of the pandemic on the HS.

#### Pre-covid global explainability

**Fig 7:**
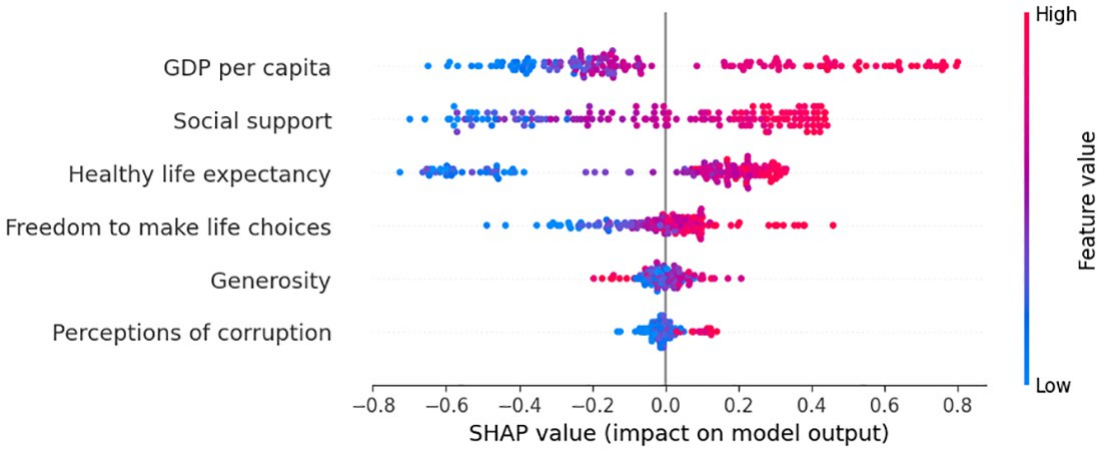
Global explanation using SHAP value plot.

**Fig 8:**
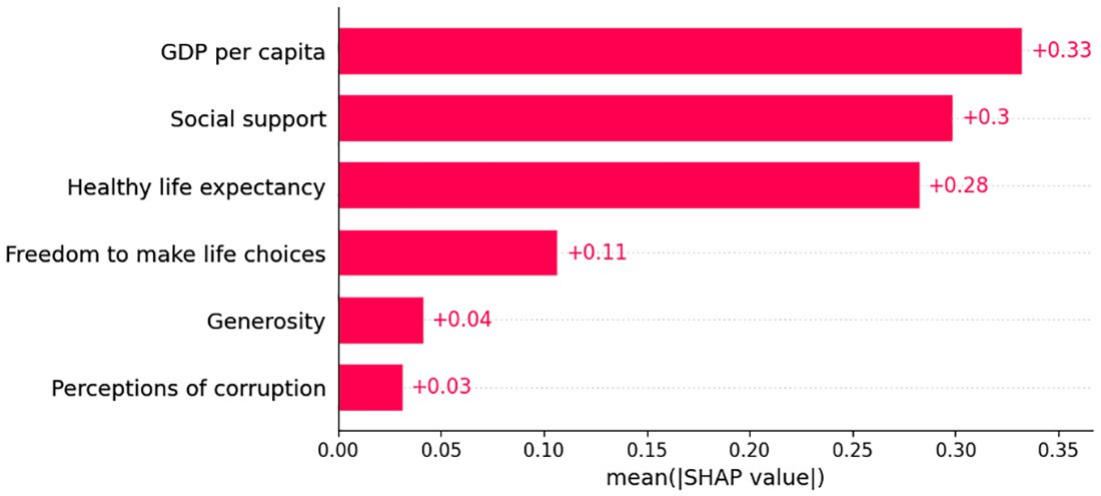
Global explanation using SHAP bar plot.

We apply SHAP as a global explainer for the predictions generates by the superior model during the pre-COVID period, both the summary plot and bar plot indicates that ‘GDP per capita’ emerged as a significant factor influencing HS. This is followed by ‘Social Support’ and ‘Healthy Life Expectancy’. The global explainability is in Fig 7 as SHAP value plot and Fig 8 as a bar plot.

**Fig 9:**
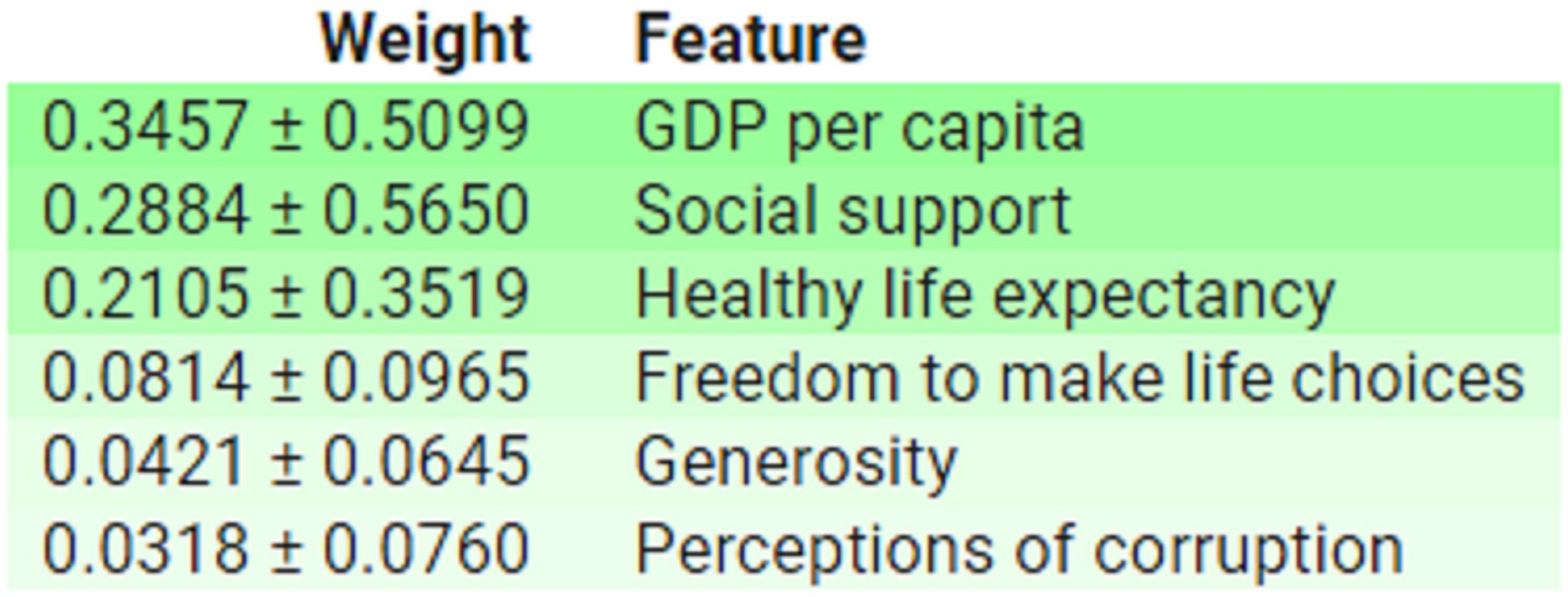
Feature importance based on ELI5 for all features.

These findings are further corroborate by permutation-based feature importance analysis conduct using ELI5, which display the average importance and standard deviation of each feature, as depict in Fig 9.

**Fig 10:**
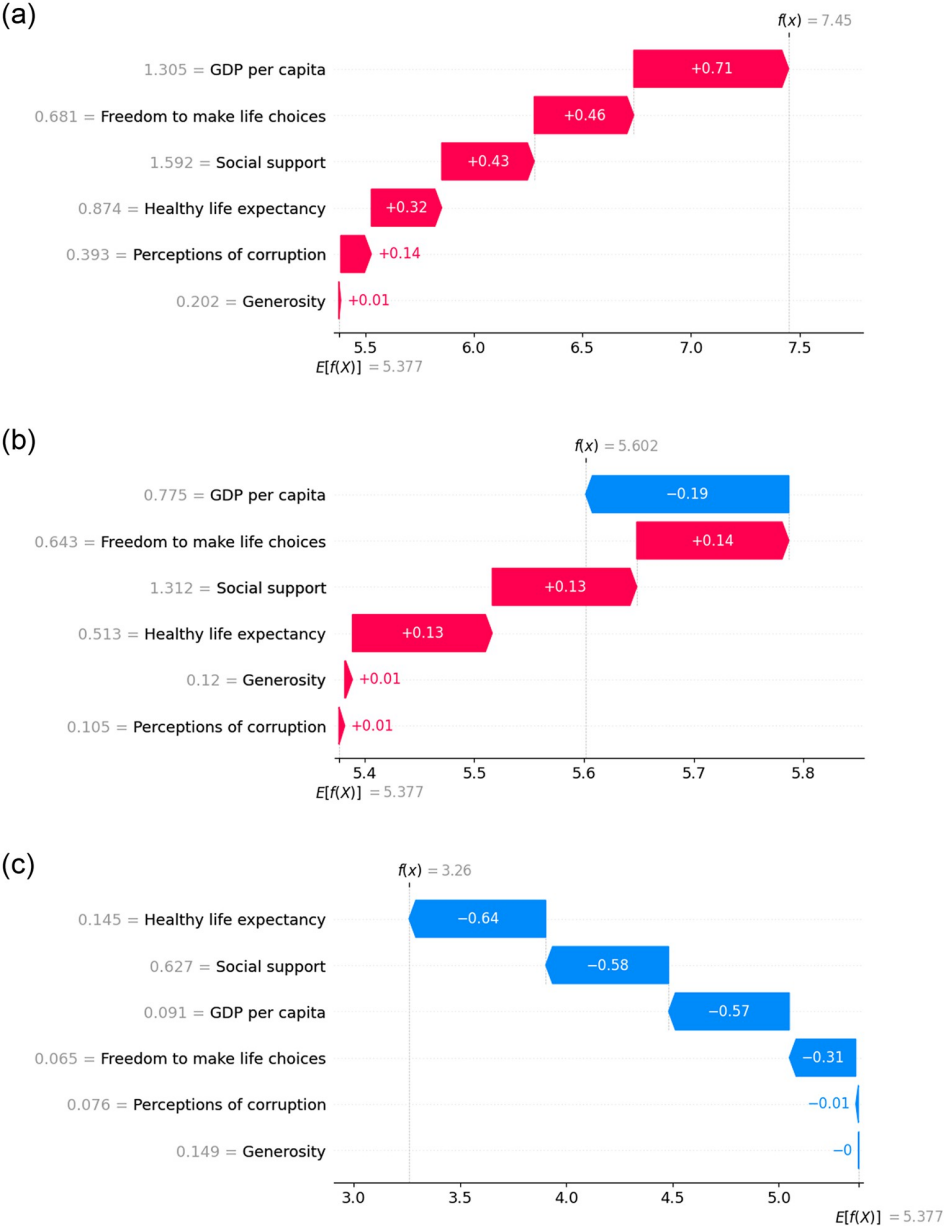
Waterfall plot representing the feature contribution for top, mid and last ranked countries before COVID.

The waterfall plot in SHAP is a visualization tool that illustrates the contribution of each feature to the predicted output of a ML model, focusing on a specific instance or observation. The waterfall plot is in Fig 10(a) show the top, in Fig 10(b) show mid, and in Fig 10(c) show the last-ranked countries during the pre-COVID period. It reveals that for the top-ranked country, each feature makes a positive contribution to its top ranking. Conversely, for the mid-ranked country, the key factor exhibits a negative contribution, while the other features contribute positively. In contrast, for the last-ranked country, all factors demonstrate a negative contribution, leading to its lowest ranking.

#### Pre-Covid local explainability

**Fig 11:**
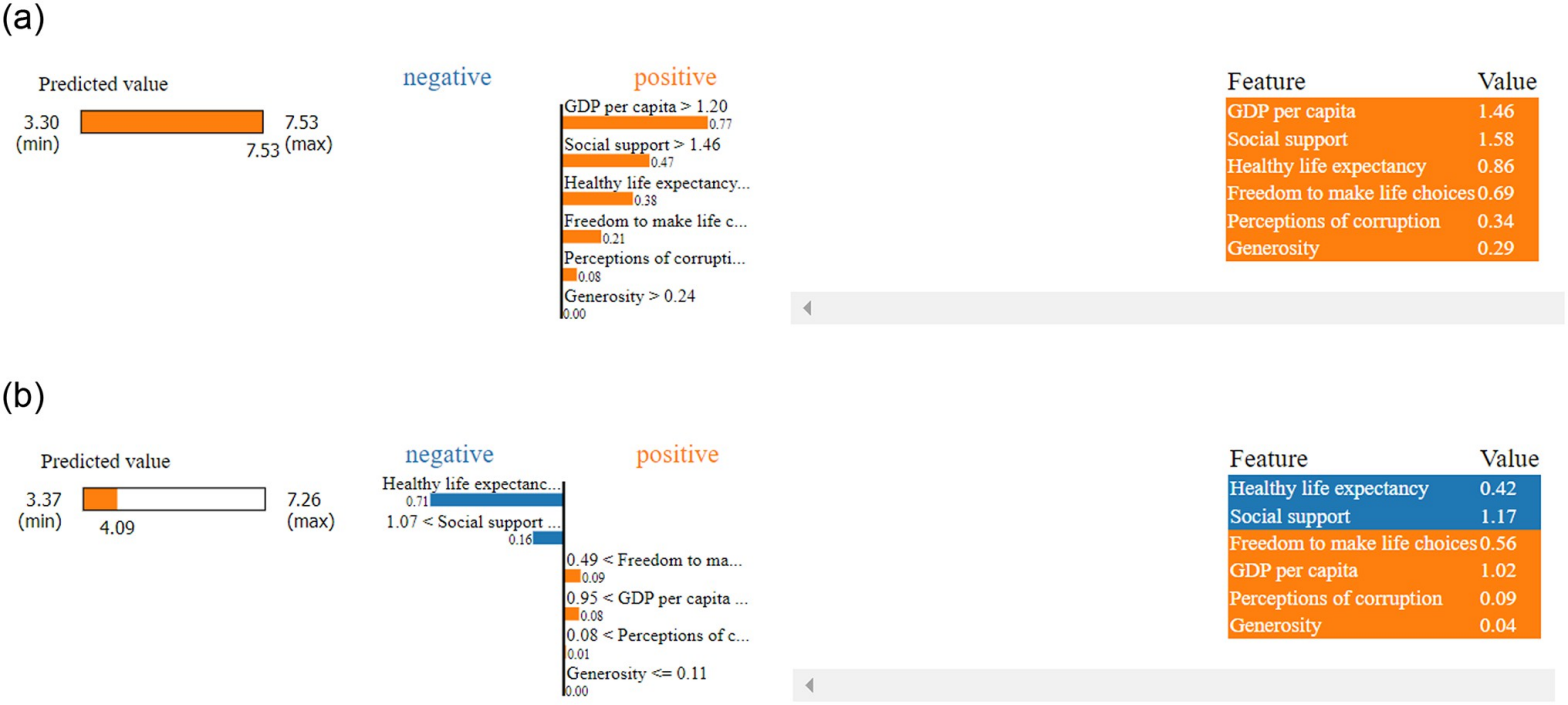
Local explanation using LIME. (a) Top ranked country, (b) Last ranked country.

Local explainability using LIME indicating for the top ranked country, in here the order of the key features are remain same and each feature positively contributed while prediciting happiness score. But for the last ranked country the order has been changed loke ‘Healthy life expectancy’ emerged as key influencer followed by ‘Social support’ and they contributed negatively to the prediction as shown in Fig 11.

#### During Covid global explainability

**Fig 12:**
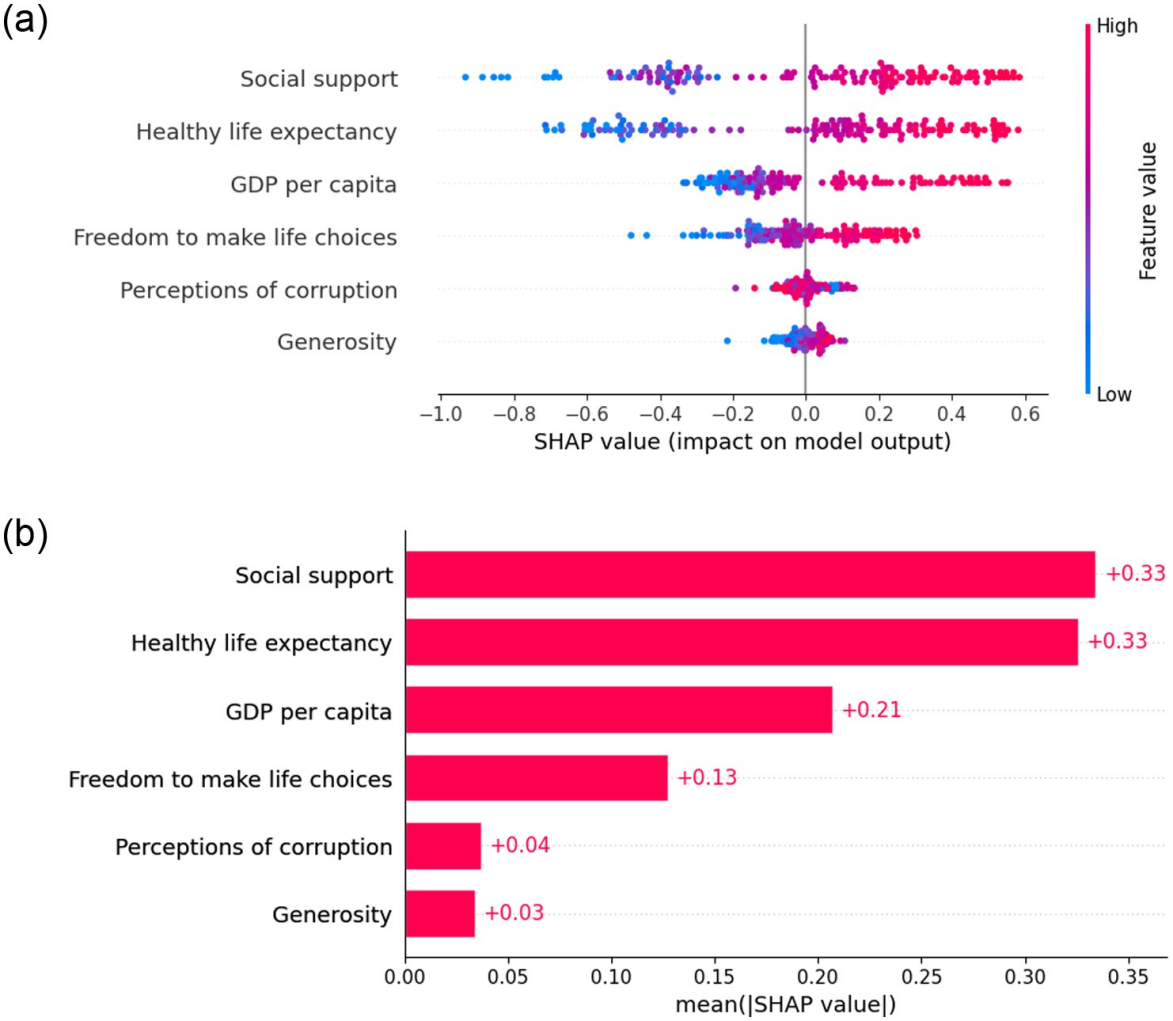
Global explanation using SHAP bar plot. (a) Global explanation using SHAP value plot. (b) Last ranked country.

During the pandemic, ‘social support’ emerged as the primary factor influencing the HS of countries, as indicated by both the SHAP summary plot and bar plot depicted in Fig 12(a) and 12(b) respectively. Furthermore, ‘healthy life expectancy’ and ‘GDP per capita’ also emerged as significant factors following social support during the COVID-19 period.

**Fig 13:**
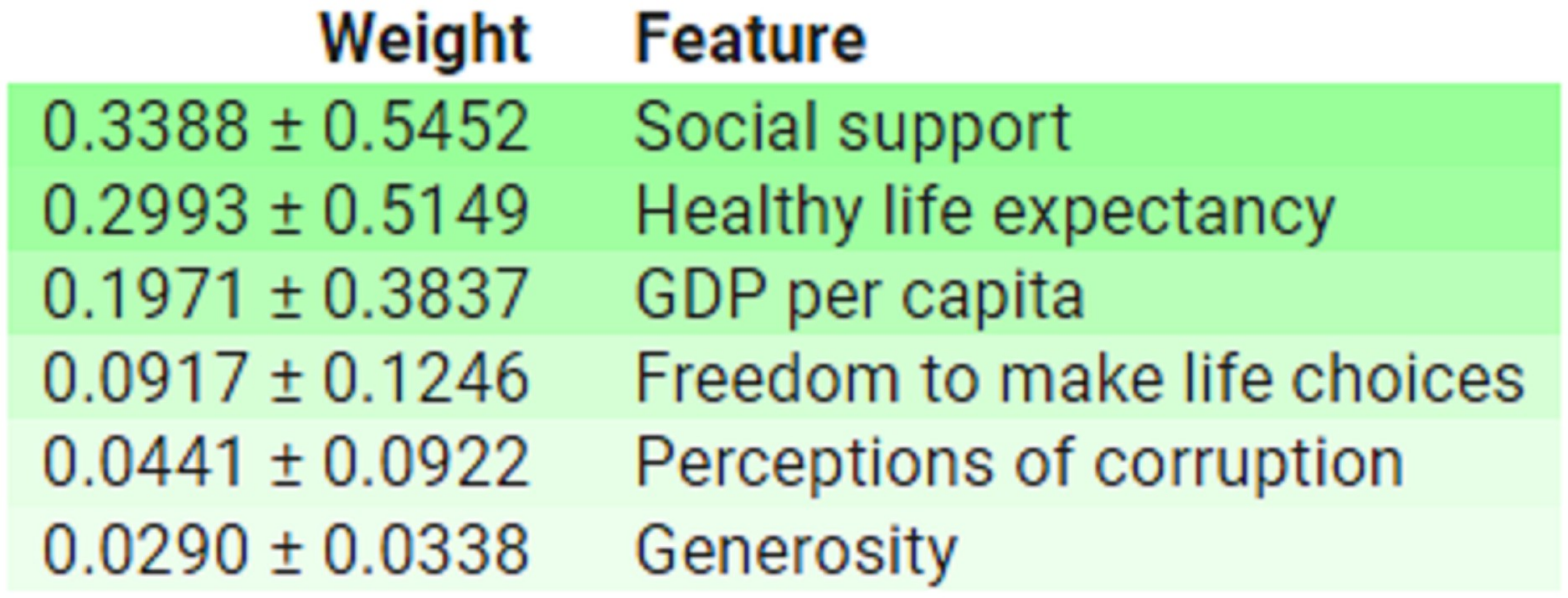
Feature importance based on ELI5 for all features.

These results supports by permutation-based feature importance analysis conduct using ELI5, as depict in Fig 13.

**Fig 14:**
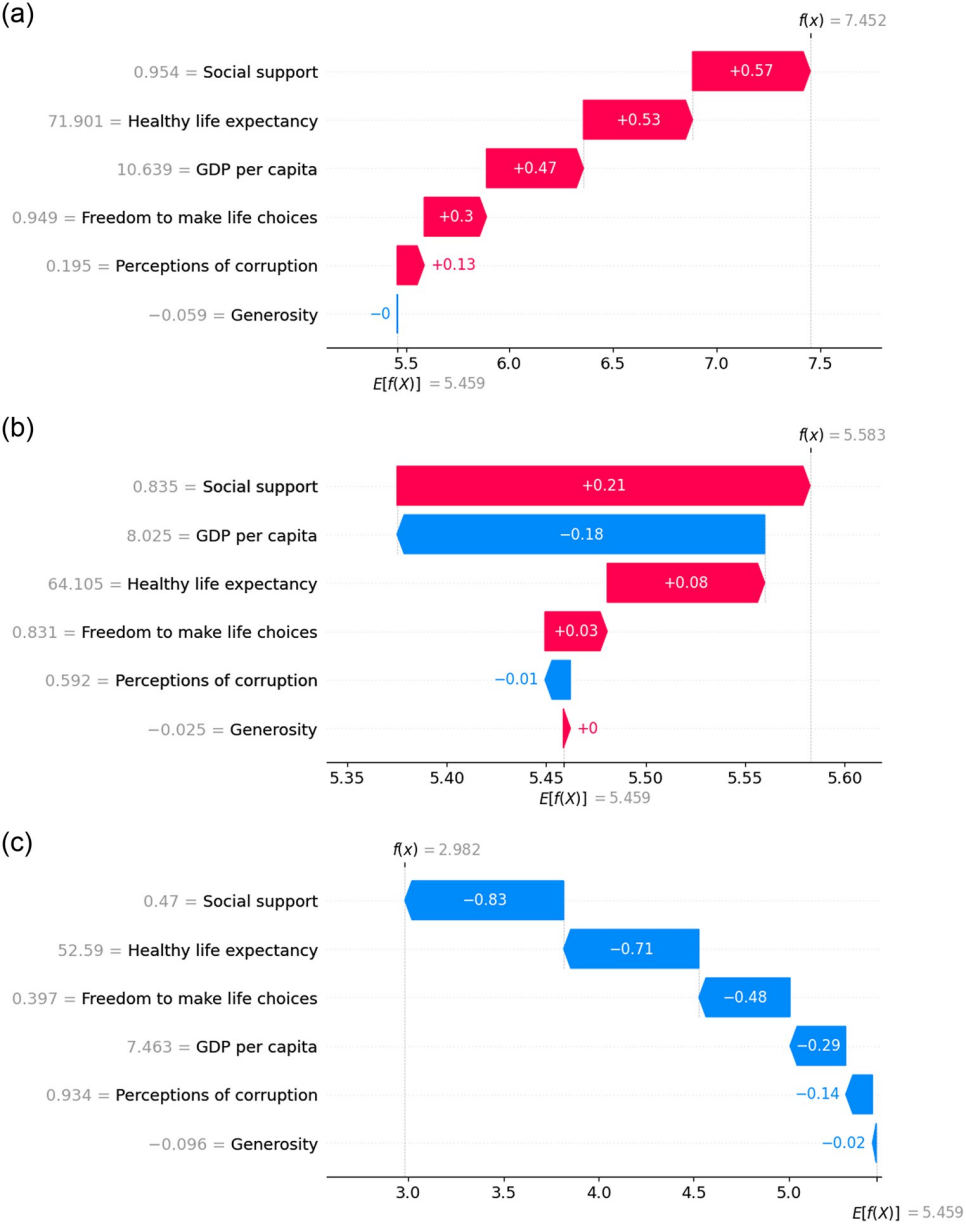
Waterfall plot representing the feature contribution for top, mid and last ranked countries during the time of COVID.

Fig 14 exhibits the waterfall plot illustrating the top, mid, and last-ranked countries. It indicates that for the top-ranked country, every feature makes a positive contribution except for generosity. In contrast, GDP per capita and perception of corruption contribute negatively, while the remaining features contribute positively for the mid-ranked country. Finally, for the last-ranked country, each feature contributes negatively when predicting the HS.

#### During Covid local explainability

**Fig 15:**
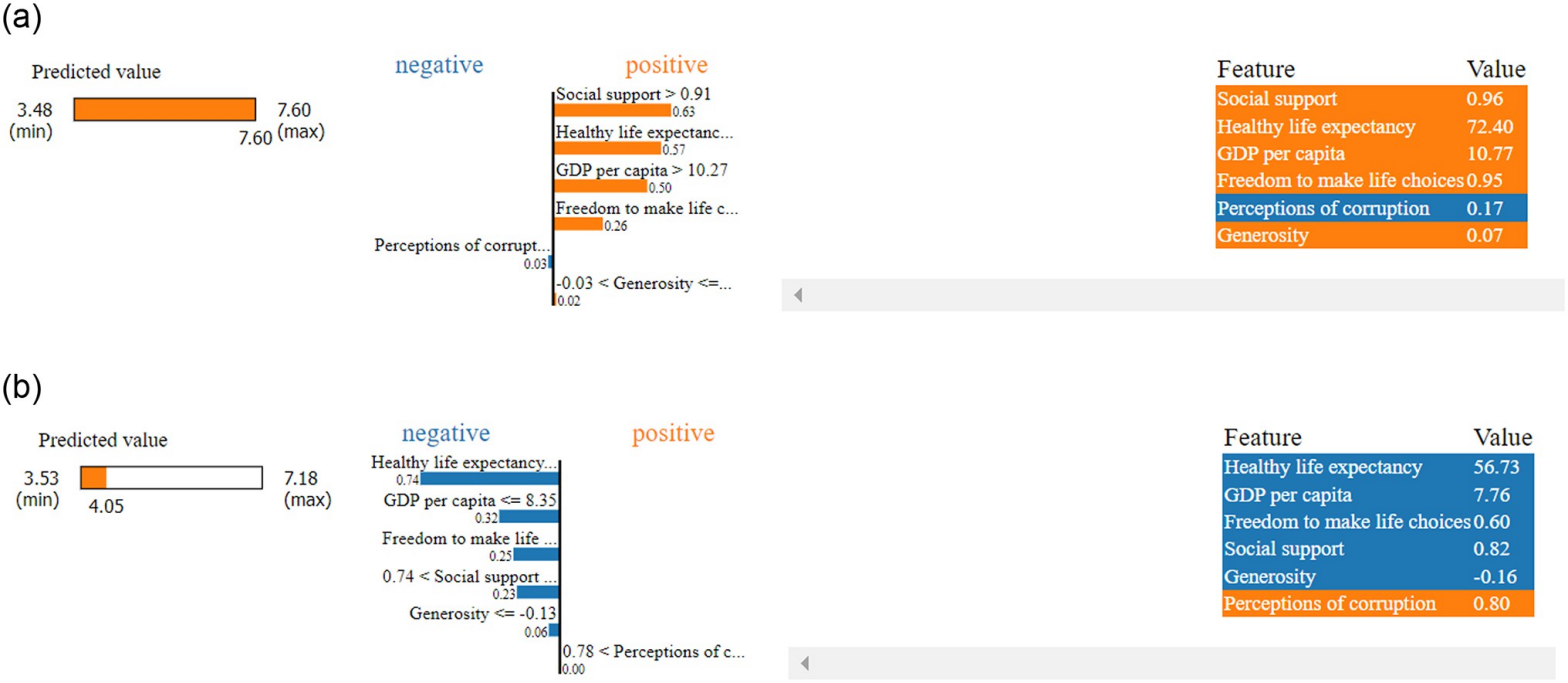
Local explanation using LIME. (a) Top country, (b) Last country.

During the pandemic session for the top-ranked country LIME explore ‘Social support’ as a key factor followed by ‘Healthy life expectancy’ and ‘GDP per capita’, and ‘perception of corruption’, where every factor contribute positively to the prediction. For the last ranked country, the key factor changed to ‘Healthy life expectancy’ and only ‘perception of corruption’ contribute positively which represent in Fig 15.

#### Post-Covid global explainability

**Fig 16:**
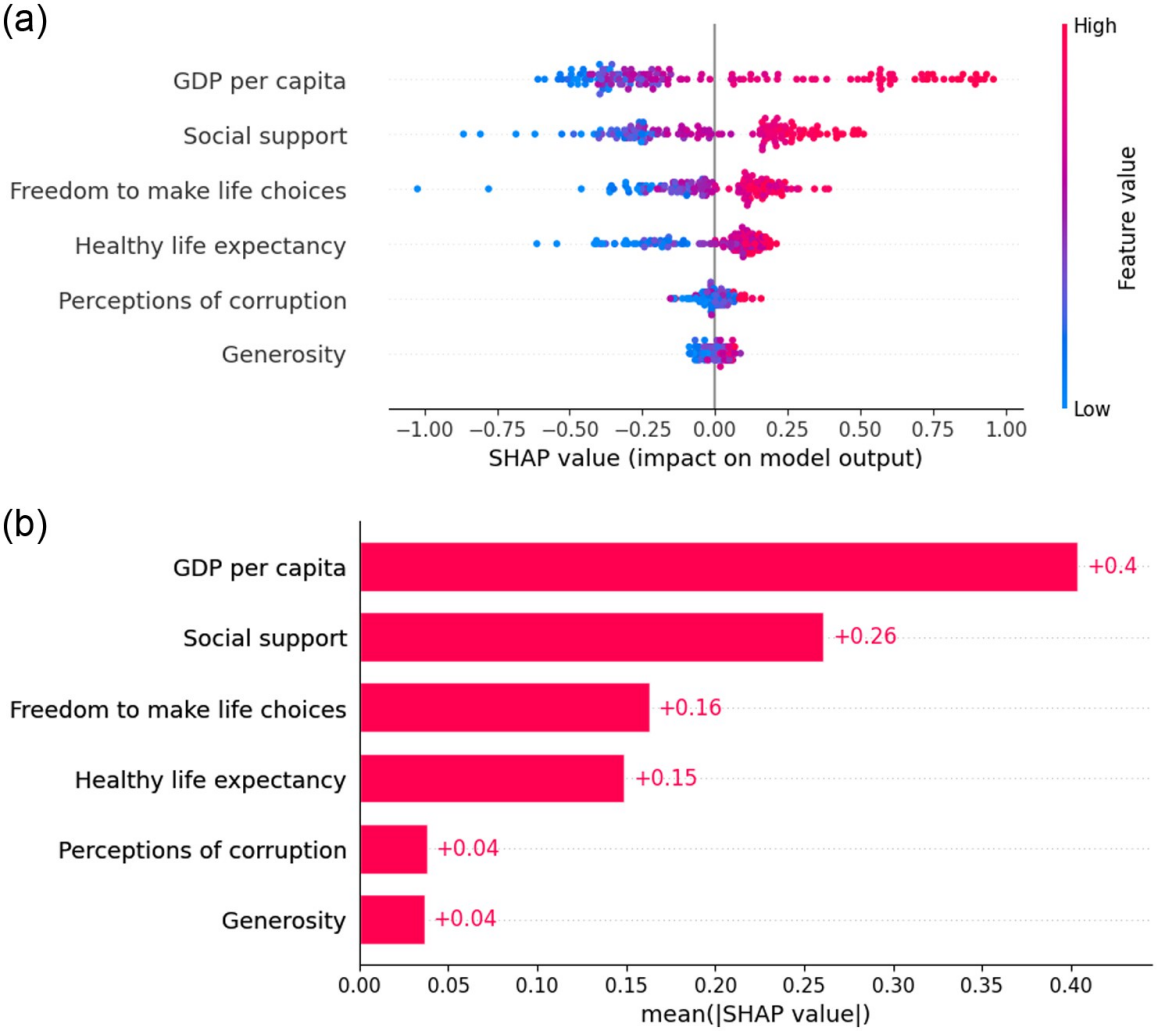
Global explanation using SHAP bar plot. (a) Global explanation using SHAP value plot. (b) Last ranked country.

Following the pandemic, ‘GDP per capita’ reclaimed its top position in influencing HS, with ‘social support’ and ‘freedom to make life choices’ emerging as the second and third influencing factors, respectively. These findings highlights using both the SHAP summary plot and bar plot depict in Fig 16(a) and 16(b) respectively.

**Fig 17:**
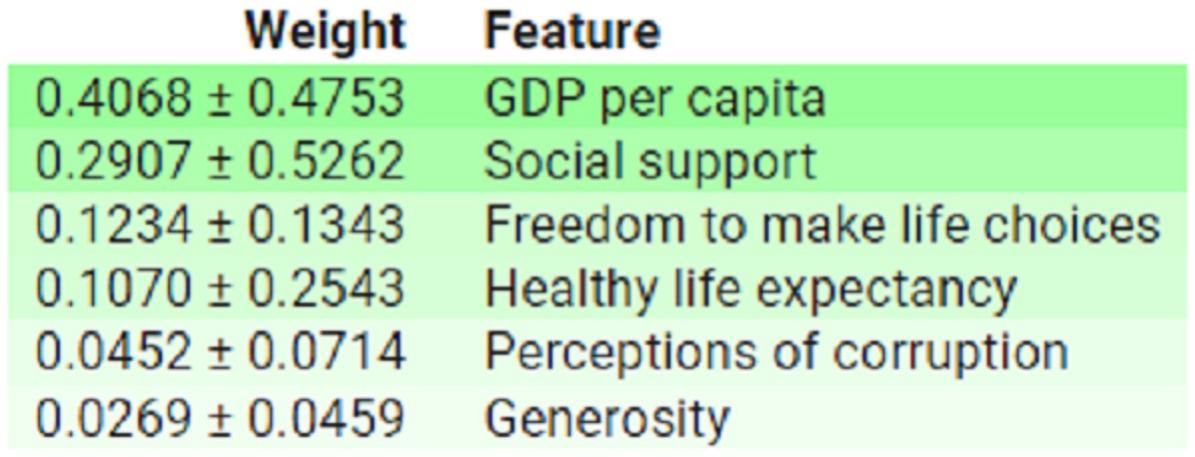
Feature importance based on ELI5 for all features.

Furthermore, permutation-based feature importance analysis conduct using ELI5, as illustrat in Fig 17, also corroborates these results.

**Fig 18:**
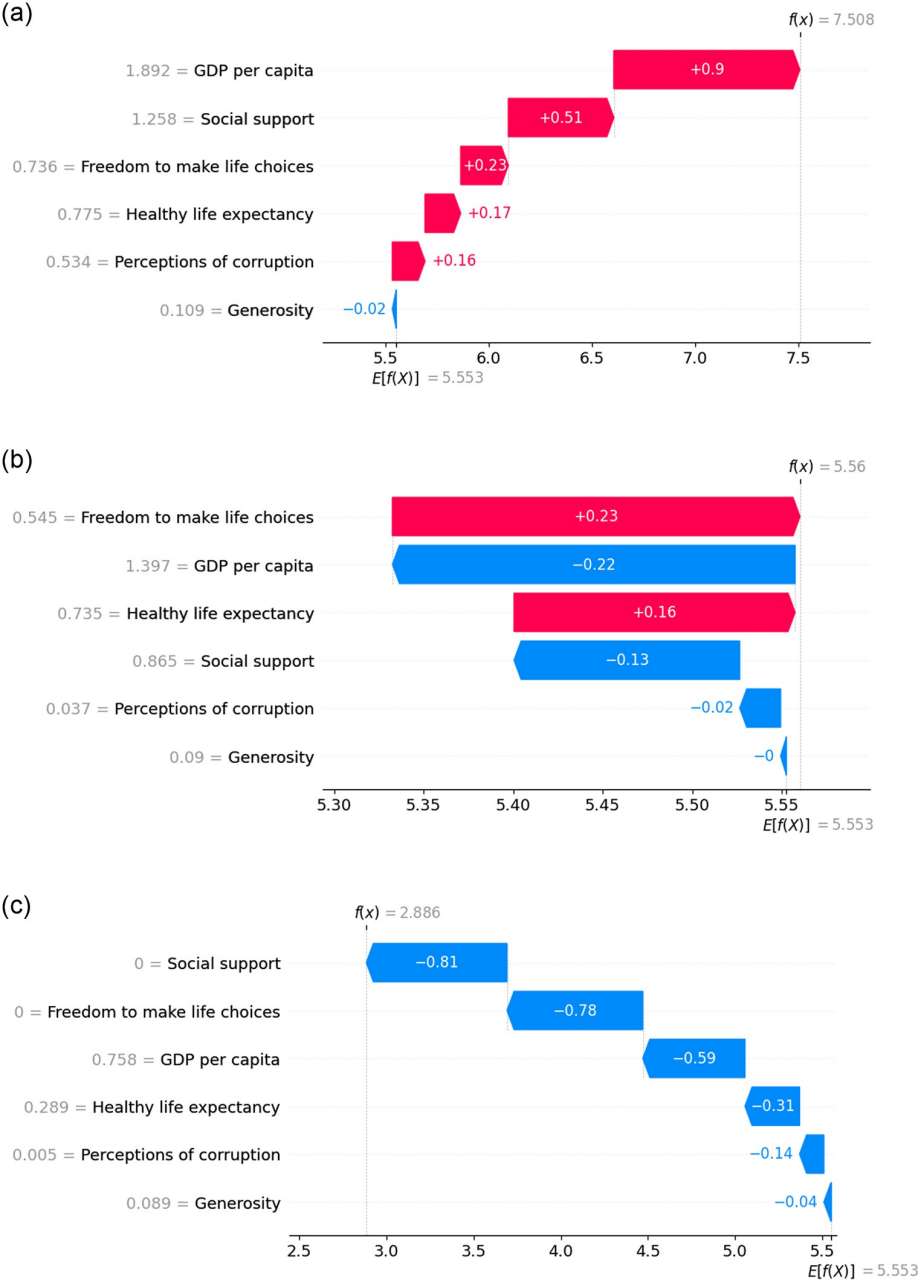
Waterfall plot representing the feature contribution for top, mid and last ranked countries after COVID.

The waterfall plots display in Fig 18 illustrates the feature importance of individual countries, including the top, mid, and last-ranked countries. For the top-ranked country, all factors except ‘Generosity’ made positive contributions. In contrast, ‘freedom to make life choices’ and ‘healthy life expectancy’ positively contributed, while the rest had negative contributions for the mid-ranked country. Conversely, after the pandemic period, all features exhibit negative contributions for the last-ranked country.

#### Post-Covid local explainability

**Fig 19:**
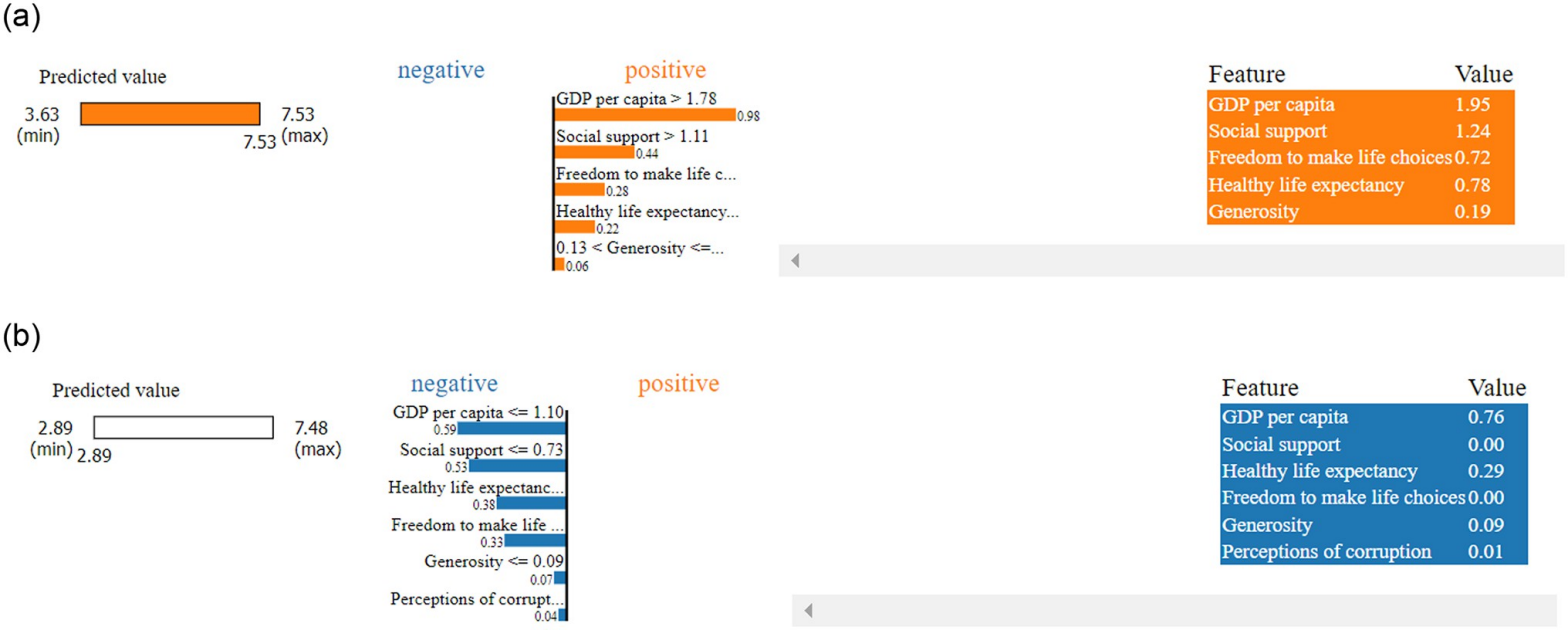
Local explanation using LIME. (a) Top ranked country. b) Last ranked country.

After pandemic session for the top ranked country LIME exploring GDP per capita as key factor followed by social support and healthy life expectancy also every factors contributed positively to the prediction. For the last ranked country the order of factors was same but every feature contributed negatively which represented in Fig 19.

### Discussion

Analyzing the performance of the model predicting the HS of countries reveals that the blending ensemble RGMLL model, which combines RF, GBM, MLP, LSTM, and LR, demonstrates the best performance across all indicators, including 0.85 *R*^2^, 0.15 MSE, and 0.38 RMSE. Overall, ensemble models such as LRGR and RGMLL outperforms individual ML and DL models. This suggests that the synergy from combining different models surpasses the performance of individual models. This finding aligns with previous research indicating the superiority of existing ensemble models [62, 63]. Among single models, RF and GP regressor exhibit the best performance. Although ensemble models compose of various model combinations outperform single models, the performance of the ensemble model varies depending on the specific algorithms combined.

The contribution of each variable to the prediction measures in the final model using several XAI methods, including SHAP, LIME, and ELI5. Before the COVID pandemic, SHAP and ELI5 reveal that the order of features contributing to the world happiness ranking was ‘GDP per capita’, ‘social support’, ‘healthy life expectancy’, ‘freedom to make life choices’, ‘generosity’, and ‘perceptions of corruption’. During the pandemic, the order changed to ‘social support’, ‘healthy life expectancy’, ‘GDP per capita’, ‘freedom to make life choices’, ‘perceptions of corruption’, and ‘generosity’. It clearly defines that ‘social support’ was the crucial and important for happiness during the pandemic time. After the COVID period, the order revert to its previous ranking as ‘GDP per capita’, ‘social support’, ‘freedom to make life choices’, ‘healthy life expectancy’, ‘perceptions of corruption’, and ‘generosity’. However, local explanations indicate that this order can vary for low-ranked countries. Finland continues to hold the top spot in the rankings for the seventh year in a row by ensuring strong social support, an effective healthcare system, and a balanced work-life environment. Countries with lower rankings can utilize the system proposed in our study to pinpoint the factors they need to enhance to climb higher in the happiness rankings.

## Conclusion

This research introduces two ensemble models, one utilizing the stacking method and the other using the blending method, to improve the predictive accuracy of national HS using data from the WHR spanning 2018-2023. Four types of models are employed for prediction that are ML,DL, stacking, and blending ensemble model. The findings reveal that individual ML and DL models perform adequately as base models for the ensemble, with the RF and MLP models outperforming other single ML and DL models, respectively. To enhance generalizability and performance, the study combines the outputs of these individual models as inputs to the proposed ensemble models. Three evaluation criteria are used to assess model performance. The results indicate that the blending ensemble model, which combines both DL and ML models, generally achieves better predictive results than the stacking ensemble model, which combines only single ML models. Furthermore, comparing the proposed blending model with traditional stacking ensemble models demonstrates the former’s significant superiority and improved generalization capability. The XAI models use to capture logical insights by deriving information from the trained models. This paper aims to find the factors influencing countries’ happiness rankings based on the input values under the criteria as per the dataset and to determine if there is any change in the order of contributing factors due to the COVID pandemic. The XAI models LIME, SHAP, and ELI5 are employed to identify the major contributing factors from the given set of features and reveal that there is a change in the order of contributing factors influencing the happiness rankings of countries. It shows that during pandemic the features ranks changes and social support was the best background of happiness. Consequently, the proposed blending ensemble model is capable of generating accurate and reliable predictions for HS. However, despite its promising performance, the proposed model has certain limitations. Specifically, the model’s superior predictive performance can be compromised by the inclusion of underperforming models in the ensemble. Thus, there is a need to develop more effective methods for combining these models to mitigate this issue in further work.
